# WLS inhibits melanoma cell proliferation through the β-catenin signalling pathway and induces spontaneous metastasis

**DOI:** 10.1002/emmm.201201486

**Published:** 2012-11-06

**Authors:** Pei-Tzu Yang, Jamie N Anastas, Rachel A Toroni, Michi M Shinohara, Jamie M Goodson, Anja K Bosserhoff, Andy J Chien, Randall T Moon

**Affiliations:** 1 Department of Pharmacology, Howard Hughes Medical Institute, Institute for Stem Cell and Regenerative Medicine, University of Washington School of MedicineSeattle, WA, USA; 2 Division of Dermatology, Department of Medicine, University of Washington School of MedicineSeattle, WA, USA; 3 Department of Biology, University of WashingtonSeattle, WA, USA; 4 Department of Pathology, University of RegensburgRegensburg, Germany

**Keywords:** beta-catenin, melanoma, spontaneous metastasis, WNTLESS, WNT secretion

## Abstract

Elevated levels of nuclear β-catenin are associated with higher rates of survival in patients with melanoma, raising questions as to how ß-catenin is regulated in this context. In the present study, we investigated the formal possibility that the secretion of WNT ligands that stabilize ß-catenin may be regulated in melanoma and thus contributes to differences in ß-catenin levels. We find that WLS, a conserved transmembrane protein necessary for WNT secretion, is decreased in both melanoma cell lines and in patient tumours relative to skin and to benign nevi. Unexpectedly, reducing endogenous WLS with shRNAs in human melanoma cell lines promotes spontaneous lung metastasis in xenografts in mice and promotes cell proliferation *in vitro*. Conversely, overexpression of WLS inhibits cell proliferation *in vitro*. Activating β-catenin downstream of WNT secretion blocks the increased cell migration and proliferation observed in the presence of *WLS* shRNAs, while inhibiting WNT signalling rescues the growth defects induced by excess WLS. These data suggest that WLS functions as a negative regulator of melanoma proliferation and spontaneous metastasis by activating WNT/β-catenin signalling.

## INTRODUCTION

WNTs comprise a family of secreted glycoproteins required for embryonic development and for tissue homeostasis in adults (Chien et al, 2009a; Clevers & Nusse, 2012). Interactions between WNT ligands and transmembrane receptors promote the accumulation of β-catenin (encoded by *CTNNB1*) in the nucleus, where it interacts with members of the TCF/LEF family of HMG-box transcription factors to promote the transactivation of target genes. For example, complexes of ß-catenin and TCF/LEF directly bind to upstream transcription regulatory elements of the *micropthalmia transcription factor* (*MITF*) gene during embryonic development, thereby instructing premigratory neural crest cells to differentiate into melanocytes (Bachmann, 2005; Dorsky et al, 1998; Larue et al, 2003; Wu et al, 2003). Wnt/β-catenin signalling regulates mouse pigmentation during development by determining the number of melanoblasts, precursor cells of melanocytes (Luciani et al, 2011). In adults, high levels of β-catenin are expressed in the cytoplasm and nucleus of benign nevi (Bachmann, 2005), which are spatially associated with approximately 20% of cutaneous melanomas and considered to be direct melanoma precursors (Luciani et al, 2011; Massi et al, 1999; Smolle et al, 1999; Urso et al, 1991).

Benign nevi express high levels of WNT transcripts including *WNT2*, *WNT5A*, *WNT5B*, *WNT7B* and *WNT10B*. These WNT ligands are also highly expressed in melanomas characterized by small and uniform cells, while WNTs are heterogeneously expressed in melanomas characterized by large, pleomorphic cells (Pham et al, 2003). Although high levels of WNT2, WNT7B and WNT10B are reported to activate WNT/β-catenin signalling (Cawthorn et al, 2011; Cheng et al, 2008; Pu et al, 2009), several studies report a reduction in nuclear β-catenin staining in high-risk melanoma patients (Chien et al, 2009b; De Panfilis et al, 2009; Kageshita et al, 2001; Maelandsmo et al, 2003). These data raise the possibility that WNT/β-catenin signalling is downregulated in melanomas associated with poor clinical outcomes independently of WNT expression.

In many cancers, the activation of WNT/β-catenin signalling occurs primarily through mutations in the pathway components (reviewed by Reya & Clevers, 2005). However, these mutations are rare in melanoma. In one study, mutations in *β-catenin* were observed in only 2 of 37 total melanoma cases, and mutations in *APC*, a negative regulator of β-catenin signalling, were observed in only 1 of 37 cases (Reifenberger et al, 2002). Furthermore, no reported mutations in the Wnt/ß-catenin pathway might account for the reduction of nuclear β-catenin that is observed in high-risk melanoma patients (Chien et al, 2009b). These observations raise questions regarding the mechanisms that underlie the observed range of nuclear ß-catenin levels in melanoma.

The modulation of WNT secretion provides a potential level of regulation of ß-catenin that has not been explored in melanoma. One of the key components required for WNT ligand secretion is *Wntless* (*WLS*), also known as *Evi*, *Sprinter* or *GPR177*. *WLS* encodes an evolutionarily conserved transmembrane protein that genetically interacts with components involved in specified secretory pathways (Harterink et al, 2011; Silhankova et al, 2010; Yang et al, 2008). Overexpressed WLS physically interacts with WNT3A, and this interaction depends upon the palmitoylation of WNT3A at Serine 209 (Coombs et al, 2010). Overexpressed WLS also interacts with VPS35, a member of the retromer complex that recycles WLS from endosomes to the trans-Golgi network, where WLS might initiate the interaction with WNT ligands (Belenkaya et al, 2008). Consistent with WLS acting in the Wnt/ß-catenin pathway, loss of function of *WLS* results in failed endoderm induction at the four-cell stage in *Caenorhabditis elegans*, a WNT-dependent phenotype (Cheng et al, 2008; Cawthorn et al, 2011; Pu et al, 2009; Thorpe et al, 1997). *Wls*-knock-out mice exhibit impaired development of the body axis, which is reminiscent of the phenotype of loss of *Wnt3* (Fu et al, 2009).

In order to better understand the mechanisms of WNT pathway regulation in melanoma, we investigated the functional consequences of increasing or decreasing WLS levels in human melanoma cells. We find that ectopic expression of WLS inhibits melanoma cell growth, while reducing levels of endogenous WLS enhances cell migration and proliferation *in vitro* and increases spontaneous lung metastasis *in vivo*. Our studies also indicate that WLS regulates cell proliferation in a β-catenin-dependent manner. These results represent the initial identification of a functional role for WLS in melanoma.

## RESULTS

### *WLS* transcripts are decreased in melanoma patient tumours

**Figure 1:**
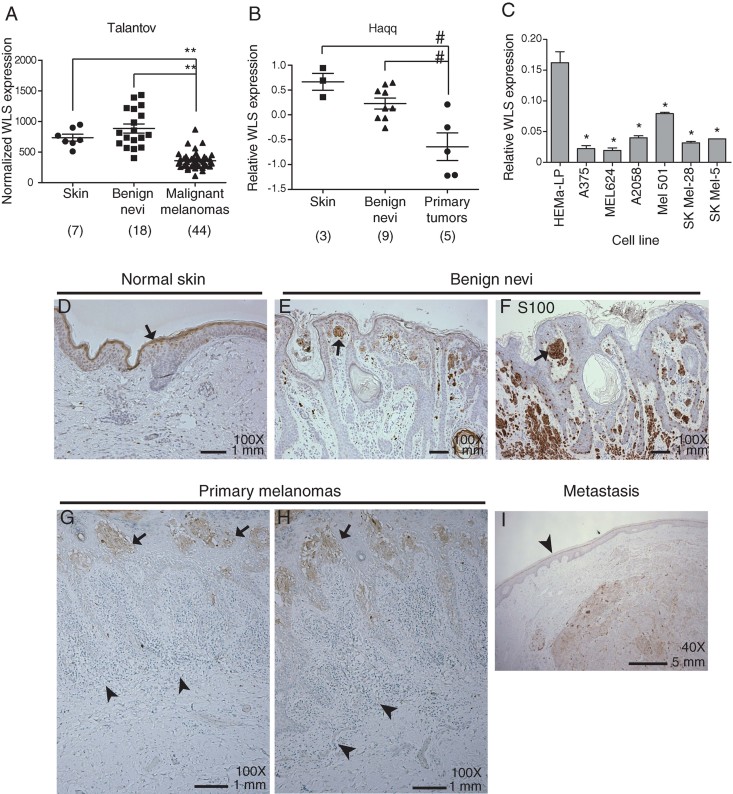
WLS expression is reduced in malignant melanomas **A.** *WLS* mRNA expression (probe# 221958_s_at) in the microarray dataset from Talantov et al (2005) including 7 normal skin, 18 benign nevi and 44 malignant melanoma samples, **p < 0.0001. Tumour samples were comprised of more than 95% tumour cells.**B.** Normalized *WLS* expression in the Haqq et al microarray dataset (2005) including 3 normal skin, 9 benign nevi and 5 malignant melanoma samples. *WLS* expression was quantified using an independent probe (probe#1AA069519), #*p* < 0.05. Tumour samples were comprised of more than 95% tumour cells.**C.** Relative *WLS* mRNA expression normalized to *GAPDH* in cultured melanocytes (HEMa-LP) and multiple human melanoma cell lines, *p < 0.01.**D–F.** Representative images of serial sections of normal human skin stained for WLS (**D** and **E**) and the melanocyte marker, S100 (**F**). (**E**) WLS staining (brown colour) is localized to the intraepidermal nevomelanocytic nests indicated by the arrow. (**F**) S100 staining is localized to regions overlapping with the WLS positive regions in serial sections as indicated by the arrow.**G,H.** Representative images of cutaneous melanomas stained for WLS. WLS is present in the intra-epidermal region (indicated by arrows) but not in invasive cells (indicated by arrowheads).**I.** Representative image of a metastatic melanoma tumour stained for WLS. Lack of WLS staining is indicated by the black arrowhead.

As a first step in evaluating whether WNT secretion has any role in melanoma, we analysed the expression of *WLS* in microarray datasets profiling gene expression patterns in melanoma patient samples (Oncomine™, Compendia Bioscience, Ann Arbor, MI). In the largest dataset (Talantov et al, 2005), the expression of *WLS* is reduced in cutaneous melanomas (*n* = 44) compared to normal skin (*n* = 7; *p* = 4.15 × 10^−5^) and reduced in melanomas compared to melanocytic, benign skin nevi (*n* = 18; *p* = 3.27 × 10^−9^; Fig 1A). In another microarray dataset (Haqq et al, 2005), *WLS* expression levels measured using an independent probe are also decreased in primary tumour samples (*n* = 5) compared to benign nevi (*n* = 9; *p* = 0.0045) and are reduced in comparison to normal skin (*n* = 3; *p* = 0.0156; Fig 1B). We also analysed a third microarray dataset (Riker et al, 2008) and observe a trend towards reduced *WLS* expression in primary melanomas (*n* = 14) compared to normal skin (*n* = 4), though the difference does not reach statistical significance (*p* = 0.061). To extend these findings, we compared the expression of endogenous *WLS* transcripts in cultured human primary melanocytes (HEMa-LP) and in several human melanoma cell lines. Quantitative real-time PCR (qPCR) reveals that *WLS* transcripts are relatively lower in multiple human melanoma cell lines compared with levels of *WLS* transcripts in melanocytes (*p* < 0.01, Fig 1C), supporting our initial finding that *WLS* expression is reduced in melanoma patient samples.

### WLS protein is expressed in benign nevi but is reduced in malignant melanoma

In order to detect endogenous WLS in patient samples, we generated a polyclonal antibody against amino acids TEMAHERVPRKLK of the WLS N-terminus in guinea pigs (see Materials and Methods Section and Supporting Information Fig S1A). We first analysed WLS expression by immunohistochemistry (IHC) in samples of normal human skin and observed that WLS is expressed in a majority of the benign nevi analysed (9 of 11 samples; Fig 1E and Supporting Information Table S1). WLS staining in normal skin is localized in the upper spinous and granular layers of the epidermis (*n* = 3; Fig 1D, indicated by arrow) as well as in intraepidermal nevomelanocytic nests (*n* = 6; Fig 1E, indicated by arrow). We also observe that the regions staining positive for WLS overlap with the melanocyte marker S100 in adjacent serial sections (Fig 1F, indicated by arrow) suggesting that WLS protein is expressed in melanocytes, but either weakly expressed or absent in other cell types present in normal human skin.

We next analysed the expression of WLS in primary melanoma patient tumours and observe positive staining for WLS in only 9 of 18 samples (Supporting Information Table S1) suggesting that WLS staining is absent in a subset of melanoma tumours. Although the difference in the proportions of nevi and melanomas expressing WLS does not reach statistical significance (Fisher's Exact, *p* = 0.1255) due to the limited sample size, these results are consistent with our finding that expression of *WLS* transcripts is reduced in melanoma samples in comparison to benign skin tissues (Fig 1A and B). In the patient specimens staining positively for WLS, we observe heterogeneous WLS staining patterns within different regions of the tumours (Fig 1G and H). Interestingly, WLS staining is readily seen in the intraepidermal regions of the tumours (indicated by arrows), yet is reduced in invasive dermal cells (indicated by arrowheads; Fig 1G and H) suggesting that WLS is reduced along the invasive front of primary melanomas.

We also analysed WLS expression in two metastatic melanoma samples but were unable to detect any signal in the dermal region of these tumours (indicated by arrowheads, Fig 1I). However, we did observe strong WLS staining at both vascular smooth muscle and pilar smooth muscles in the same section (indicated by arrow, Supporting Information Fig S1C, left panel), which we verified by staining for a smooth muscle marker, smooth muscle actin (SMA), in adjacent serial sections (Supporting Information Fig S1C, right panel). A more detailed analysis of our IHC data obtained from the 11 benign nevi, 18 primary melanomas and 2 metastatic tumours included in this study can be found in the Supporting Information Table S1. These qualitative studies of WLS protein levels in patient samples, taken together with our analysis of *WLS* transcript expression, support the hypothesis that WLS is reduced in melanoma.

### Reducing endogenous WLS promotes spontaneous metastasis of melanoma *in vivo*

Since WLS levels are reduced in melanoma samples, we wondered whether WLS might functionally contribute to melanoma growth and metastasis. To address this question, we determined the consequences of reducing endogenous WLS in a xenograft model of melanoma. We first generated human melanoma cell lines stably expressing shRNAs targeting human *WLS*. Our immunoblot analysis validates that WLS abundance is strongly reduced in A375 cells expressing two independent *WLS* shRNAs compared to non-silencing and *GAPDH*-directed control shRNAs (Supporting Information Fig S2A). To determine if WLS knockdown disrupts WNT secretion, we analysed secreted endogenous WNT5A and endogenous WNT10B in cell culture media by immunobloting. We find that *WLS* shRNAs reduce both secreted WNT5A and WNT10B in the media from A375 cells (Supporting Information Fig S2A and S2E). These data indicate that loss of WLS in melanoma cells prevents the secretion of multiple WNT ligands, which is consistent with previous studies (Coombs et al, 2010). Further qPCR analysis demonstrates that *WNT5A* transcripts are not altered by *WLS* shRNAs (Supporting Information Fig S2B) suggesting that loss of WLS leads to a decrease in WNT5A protein secretion but does not alter *WNT5A* expression. Occasionally, we observe elevated WLS and a corresponding increase in the levels of secreted WNT5A/B in cells transduced with *GAPDH* shRNA, however, these effects are not robust (Supporting Information Fig S2A).

**Figure 2:**
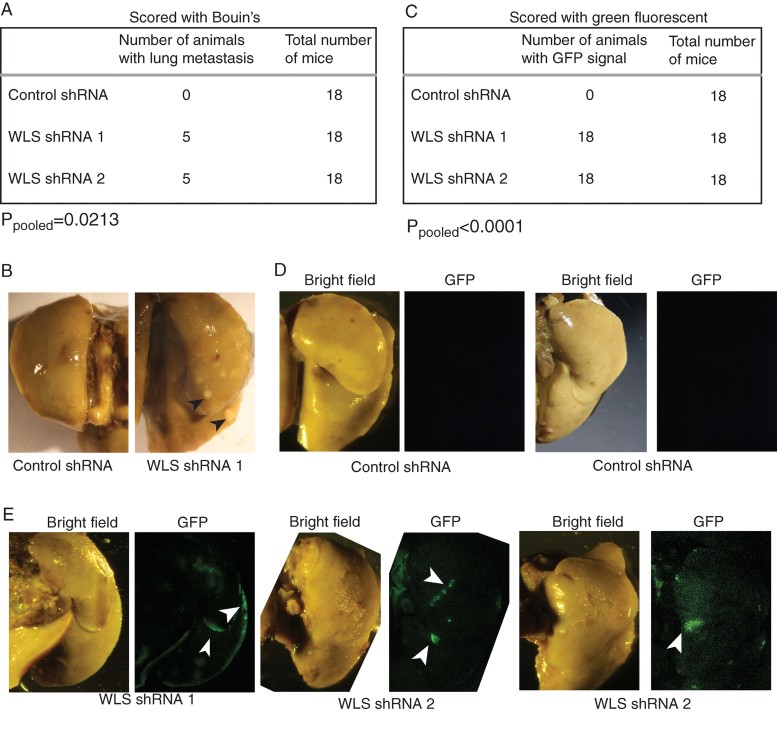
Reducing expression of WLS promotes melanoma spontaneous lung metastasis in a xenograft model Post-mortem analysis of spontaneous lung metastasis in NOG mice. Mice were subcutaneously transplanted either with 500 or 1000 A375 cells stably transduced with *WLS* or control shRNAs. **A.** Summary of spontaneous lung metastases scored by Bouin's solution staining. Fisher's exact test, *p*_pooled_ = 0.0213.**B.** Representative images of lungs from a mouse injected with *WLS* shRNA cells. Lung tissue was fixed in Bouin's solution. Black arrowheads indicate representative metastatic foci.**C.** Summary of spontaneous lung metastases scored using a fluorescent dissecting microscope to detect the GFP tracer expressed by shRNA-transduced cells. Fisher's exact test, *p*_pooled_ < 0.0001.**D,E.** Lung images taken in bright field or GFP channel with the same exposure time from mice transplanted with A375 cells expressing either control shRNA (**D**) or *WLS* shRNA (**E**). White arrowheads indicate GFP positive regions.

We then injected A375 cells expressing either control or *WLS* shRNAs subcutaneously into the flanks of NOD-SCID γ-null (NSG) mice. Analysing the resulting tumours reveals that cells stably transduced with *WLS* shRNAs exhibit a trend towards increased tumour size in xenografts (*p* = 0.0924 and 0.1364, Supporting Information Fig S3). We also examined the lungs of the animals used in this study for the presence of metastatic lesions, which express endogenous *Wls* according to the Gene Card database (http://www.genecards.org). In the present study, we detect spontaneous lung metastasis in 10 of 36 animals transplanted with *WLS* shRNA cells (Fig 2A). We detect multiple foci of metastatic colonies on the surfaces of lungs in these mice (indicated by black arrow heads, Fig 2B). In contrast, none of the 18 animals transplanted with control shRNA cells develop lung metastases (Fisher's exact test, *p*_pooled_ = 0.0231; Fig 2A) consistent with previous studies reporting that A375 cells do not form lung metastases up to 261 days post transplantation (Graells et al, 2004; Kozlowski et al, 1984).

Since our shRNA vectors contain a GFP tracer, we subsequently scored the lungs of the animals for the presence of GFP-positive metastatic cells. We observe GFP-positive areas in all 36 lungs from mice xenografted with *WLS* shRNA cells, but do not detect any GFP signal in the lungs from mice xenografted with control shRNA cells (Fisher's exact test, *p*_pooled_ < 0.0001, Fig 2C–E). Some of the lungs from the mice xenografted with *WLS* shRNA exhibit microscopically detectable GFP-positive areas at multiple locations (indicated by white arrowheads, Fig 2E). These results suggest that reducing WLS promotes spontaneous melanoma metastasis *in vivo*.

Given these results, we then asked if changes in WLS expression correlate with metastasis in melanoma patients. One previous microarray study reported the expression profiles of cutaneous primary melanoma samples derived from patients with or without distant metastases within 4 years of diagnosis (Winnepenninckx et al, 2007). In the study, *WLS* expression is decreased in primary tumours from patients who developed distant metastasis (*p* = 0.0471, Supporting Information Fig S4; Winnepenninckx et al, 2007), further suggesting that reduced *WLS* expression in primary tumours correlates with the presence of metastatic disease. Interestingly, WLS expression levels are reduced in low-passage melanoma cells derived from metastatic tumours compared to cell lines derived from primary tumours (*n* = 11; Valsesia et al, 2011). However, in freshly isolated metastatic tumours, *WLS* expression does not differ from paired primary tumours in two studies (Pasmanik-Chor et al, 2011; Winnepenninckx et al, 2006; Supporting Information Table S2).

### WLS inhibits melanoma cell growth, but not cell migration or invasion

**Figure 3:**
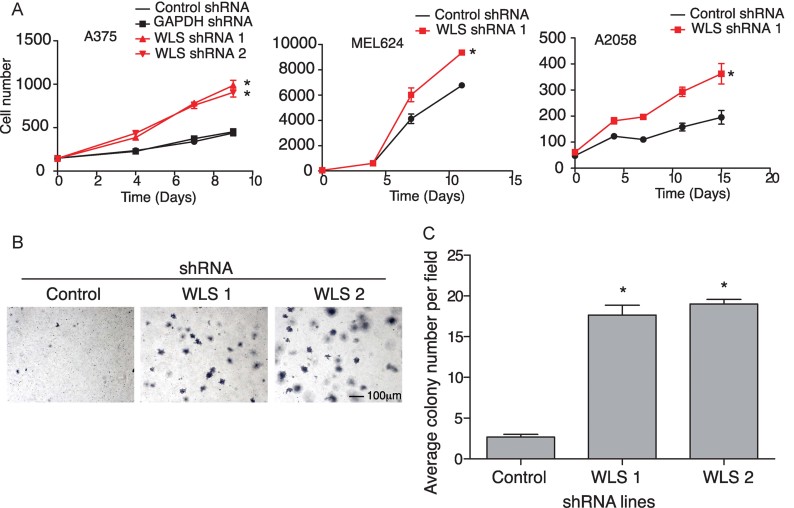
Reducing expression of WLS enhances melanoma proliferation in soft agar and in two-dimensional culture **A.** Growth curves of A375, MEL624 and A2058 cells transduced with shRNAs targeting *WLS*, *GAPDH* or a non-silencing control in two-dimensional culture.**B,C.** Soft agar assays were performed to compare the anchorage independent growth of A375 cells transduced with control or *WLS* shRNAs. (**B**) Representative photos of soft agar colonies stained with crystal violet solution 10 days after embedding cells at single cell density, *p < 0.005. (**C**) Quantification of the average number of colonies formed in soft agar, *p < 0.0005.

In addition to the ability to migrate and invade into tissue surrounding primary tumours, metastatic melanoma cells must also possess the ability to survive and proliferate in the lungs and other distal sites. Therefore, we next investigated whether WLS regulates melanoma cell proliferation. The growth of *WLS* shRNA-transduced A375 cells does not differ from control cells when plated at high density (2500 cells/cm^2^, Supporting Information Fig S3C). However, *WLS* shRNAs increase A375 cell proliferation by an average of 85.7% compared to control shRNAs when plated at low density (312 cells/cm^2^; *p* = 0.0039, Fig 3A). *WLS* shRNAs also enhance the proliferation of both A2058 (85.6%, *p* = 0.0014) and MEL624 cells (37.1%, *p* = 0.0019) compared to control and *GAPDH* shRNAs (Fig 3A) indicating that WLS negatively regulates the proliferation of multiple melanoma cell lines. We also conducted soft agar assays to determine if WLS regulates anchorage-independent growth in melanoma (Fig 3B). We quantified colony formation in this assay and find that A375 cells expressing *WLS* shRNAs form more than three times as many colonies as A375 cells expressing a control shRNA (*p* < 0.0005, Fig 3C).

We next monitored cell migration and invasive behaviour *in vitro*. We first assessed melanoma cell migration in a wound-healing assay (Rodriguez et al, 2005) but observed no difference in the migration of A375 cells transduced with either control or *WLS* shRNAs on type I collagen (Supporting Information Fig S5A). Next, we tracked the migration of individual cells using live cell imaging. For these experiments, we plated cells at low density (250 cells/cm^2^) and tracked migration for 24 h. Results from three individual experiments reveal that the average migration speed was slightly increased in A375 cells transduced with *WLS* shRNAs compared to control shRNA (Supporting Information Fig S5C). Although the increase in the rate of cell migration is statistically significant (*p* = 0.00130), the relative change in migration is very small (18 ± 12%). We next tested whether WLS regulates the invasion of melanoma cells into a type I collagen matrix using a three-dimensional spheroid model (Hattermann et al, 2011). To conduct these spheroid assays we cultured A375 cell in low-attachment plates so that they grow as spheroids and then embedded the spheroids in type I collagen. After 24 h embedded in collagen, many of the cells invade into the surrounding matrix. However, there is no significant difference in the invasion of *WLS* shRNA and control shRNA cell lines in this assay (Supporting Information Fig S5B). Together, these studies support a model in which WLS loss-of-function minimally affects cell migration and invasion *in vitro*.

### WLS regulates WNT/β-catenin-dependent transcription in melanoma

We have previously shown that WNT3A can activate β-catenin-dependent transcription and inhibit the proliferation of some melanoma cell lines (Biechele et al, 2012; Chien et al, 2009b). Since *WLS* shRNAs enhance melanoma cell growth, we hypothesized that WLS might regulate melanoma cell proliferation via the WNT/β-catenin pathway. To test this hypothesis, we first asked whether WLS regulates WNT/β-catenin signalling in melanoma cell lines using a β-catenin-activated reporter driving firefly luciferase (BAR-luciferase; Biechele & Moon, 2008). Transfecting A2058 cells with *WNT3A* leads to a 5.2-fold enhancement of BAR-luciferase, which was inhibited by siRNA-mediated knockdown of *WLS* and *VPS35* (Supporting Information Fig S2F) suggesting that WLS-dependent WNT secretion regulates WNT/β-catenin signalling in melanoma.

**Figure 4:**
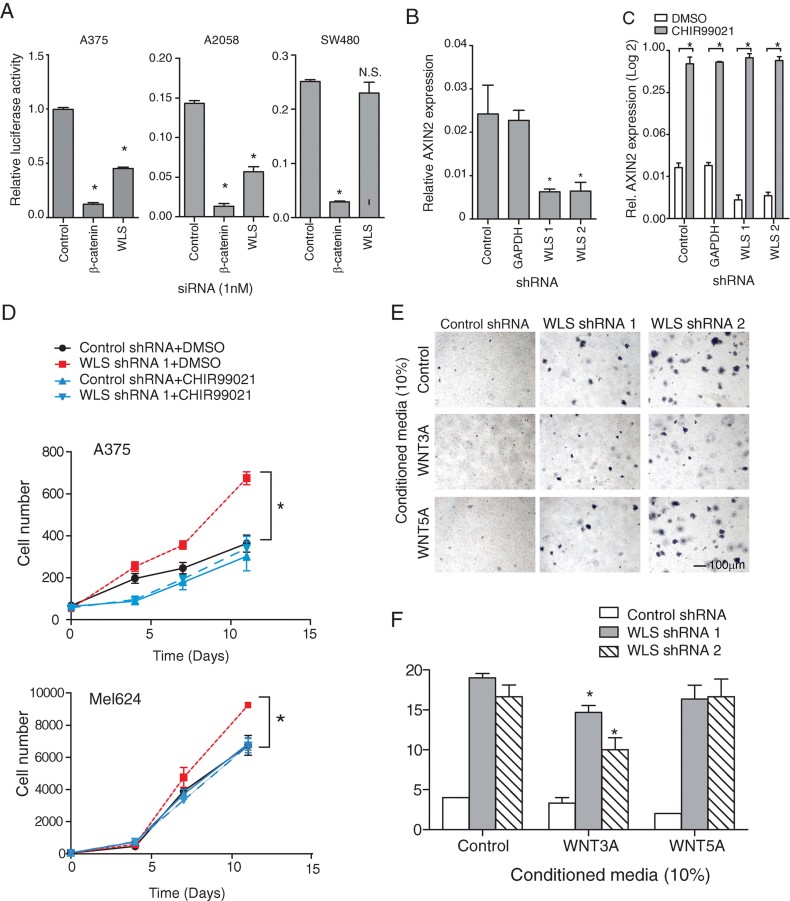
Reducing expression of WLS promotes melanoma proliferation by inhibiting WNT/β-catenin signalling **A.** Normalized WNT/β-catenin transcriptional reporter (BAR-luciferase) activity in A375, A2058 and SW480 cells transfected with siRNAs targeting *WLS*, *β*-*catenin*/*CTNNB1* or a control siRNA. BAR-luciferase values were normalized to a constitutively active renilla luciferase reporter to control for cell number, *p < 0.01.**B.** Quantification of *AXIN2* mRNA in A375 cell lines expressing shRNAs against *WLS*, *GAPDH* or control. *AXIN2* expression was normalized to *RN18S*, *p < 0.01.**C.** Quantification of *AXIN2* mRNA in A375 cell lines treated with 0.5 µM CHIR99021, a WNT/(-catenin activator or DMSO control for 72 h, Two-way ANOVA, *p* < 0.0001. Expression values are shown using a Log2 scale. *AXIN2* expression was normalized to *RN18S*.**D.** Growth curves of A375 (upper panel) and MEL624 (lower panel) shRNA cell lines treated with 0.5 µM CHIR99021 (blue) or DMSO (red and black), *p < 0.01.**E,F.** Soft agar assays were performed to compare the anchorage independent growth of A375 cells transduced with control or *WLS* shRNAs. Embedding mixture contains either 10% WNT3A-, WNT5A- or control conditioned media. (**E**) Representative photos of soft agar colonies stained with crystal violet solution 10 days after embedding cells at single cell density. (**F**) Quantification of the average number of colonies formed in soft agar, *p = 0.0147.

To determine if WLS also regulates endogenous WNT/β-catenin signalling, we analysed the activation of the BAR-luciferase reporter in A375 cells transfected with various siRNAs. As a positive control, we transfected A375 and A2058 cells with *CTNNB1* siRNA and observe almost a complete loss of BAR-luciferase activity, confirming that the transcription of the reporter construct is indeed driven by β-catenin (Fig 4A). We find that *WLS* siRNA also results in a decrease in BAR-luciferase activity relative to control siRNA in both A375 cells (*p* = 0.0091, Fig 4A, left panel) and in A2058 cells (*p* = 0.0073, Fig 4A, centre panel). These results indicate that WLS is required for endogenous WNT/β-catenin signalling in multiple melanoma cell lines. Importantly, *WLS* siRNA does not affect BAR-luciferase activity in SW480 colon cancer cells, which harbour APC mutations leading to constitutive activation of β-catenin-dependent transcription in the absence of WNT stimulation (Fig 4A, right panel). Consistent with the BAR reporter result, qPCR also reveals a corresponding down-regulation of the WNT/β-catenin target gene *AXIN2* (Jho et al, 2002) in A375 cell lines transduced with *WLS* shRNAs (*p* < 0.001, Fig 4B).

### *WLS* shRNA enhances melanoma cell proliferation by downregulating WNT/β-catenin signalling

We next asked whether *WLS* shRNA enhances melanoma growth through the inhibition of WNT/β-catenin signalling. Addition of exogenous WNT3A suppresses the increased colony-forming ability usually exhibited by *WLS* shRNA-transduced cell lines (*p* = 0.0147, images in Fig 4E and quantification in Fig 4F). We also re-activated WNT/β-catenin signalling downstream of WNT secretion using the small molecule GSK3β inhibitor CHIR99021 (Ring et al, 2003). Treatment with 0.5 µM CHIR99021 induces the expression of *AXIN2* mRNA in both control and *WLS* shRNA cells (Two-way ANOVA, *p* < 0.0001, Fig 4C). This result confirms that we can rescue β-catenin-dependent transcription downstream of endogenous WNT secretion. In both A375 and MEL624 cells, activating WNT/β-catenin signalling downstream of WNT secretion with CHIR99021 attenuates the increase in cell number induced by *WLS* shRNA (*p* < 0.01, Fig 4D). These data suggest that loss of WLS enhances proliferation by inhibiting WNT/β-catenin signalling.

### WLS overexpression reduces melanoma cell numbers by activating WNT/β-catenin signalling

**Figure 5:**
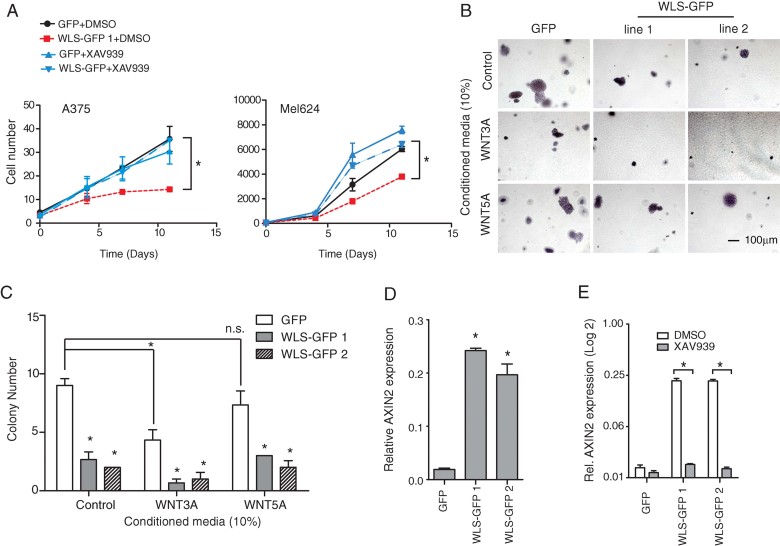
Increasing expression of WLS inhibits melanoma proliferation by activating WNT/β-catenin signalling **A.** Growth curves of A375 and MEL624 cells expressing GFP or WLS-GFP that were treated with 0.5 µM XAV939, a WNT/β-catenin inhibitor, (blue) or DMSO (red and black) in two-dimensional culture, *p* < 0.001.**B,C.** Soft agar assay of A375 cells stably expressing GFP or WLS-GFP. Cells were grown in the presence of either 10% control, WNT3A- or WNT5A-conditioned-media. (**B**) Representative photos of soft agar colonies stained with crystal violet after 3 weeks in culture. (**C**) Quantification of the average number of colonies formed, *p < 0.0001.**D.** Quantification of *AXIN2* mRNA in A375 cells expressing either GFP or WLS-GFP. *AXIN2* expression was normalized to *GAPDH*, two-way ANOVA, *p* < 0.0001.**E.** Quantification of *AXIN2* mRNA in A375 cell lines treated with the WNT/β-catenin inhibitor XAV939 (0.5 µM) or DMSO control for 72 h. *AXIN2* expression was normalized to *GAPDH*, two-way ANOVA, *p* < 0.0001.

As reduced WLS enhances melanoma cell growth, we next asked if increasing WLS inhibits melanoma cell proliferation. We established melanoma cell lines stably expressing either a WLS-GFP fusion protein (WLS-GFP) or GFP as a control. After confirming the expression of WLS-GFP by immunoblotting (Supporting Information Fig S2C), we monitored the growth of these cells in both two-dimensional culture and soft agar assays. In two-dimensional culture, ectopic expression of WLS-GFP strongly reduces cell numbers in both A375 (*p* = 0.0003) and MEL624 cells (*p* = 0.0002; Fig 5A). Cells expressing WLS-GFP also form fewer colonies compared to control cells when grown in soft agar (Two-way ANOVA, *p* < 0.0001, images in Fig 5B, quantification in Fig 5C). Together, these studies are consistent with the hypothesis that WLS negatively regulates melanoma cell proliferation *in vitro*.

Since *WLS* shRNA-transduced cells exhibit loss of WNT/β-catenin signalling, we then asked if the WNT/β-catenin pathway is activated in A375 cells upon over-expression of WLS-GFP. Indeed, the expression of *AXIN2* is elevated in cells stably expressing WLS-GFP compared to GFP control (Two-way ANOVA, *p* < 0.0001, Fig 5D). We then determined whether overexpression of WLS-GFP inhibits melanoma cell proliferation through the WNT/β-catenin pathway. To inhibit WNT/β-catenin signalling downstream of WNT secretion, we used the tankyrase inhibitor, XAV939 (Huang et al, 2009). XAV939 reduces the elevated levels of *AXIN2* seen in A375 cells expressing WLS-GFP (Two-way ANOVA, *p* < 0.0001, Fig 5E). In parallel, XAV939 also rescues the growth inhibition induced by WLS-GFP overexpression in A375 and MEL624 cells (*p* < 0.01, Fig 5A). As predicted, activating WNT/β-catenin signalling in a soft agar assay with WNT3A conditioned media further inhibits colony formation compared to control or WNT5A-conditioned media in A375 cells (pictures in Fig 5B, quantification in Fig 5C). These results together suggest that high levels of WLS inhibit melanoma proliferation by activating WNT/β-catenin signalling.

### Endogenous β-catenin signalling in melanoma cells is mediated by WNT ligands

**Figure 6:**
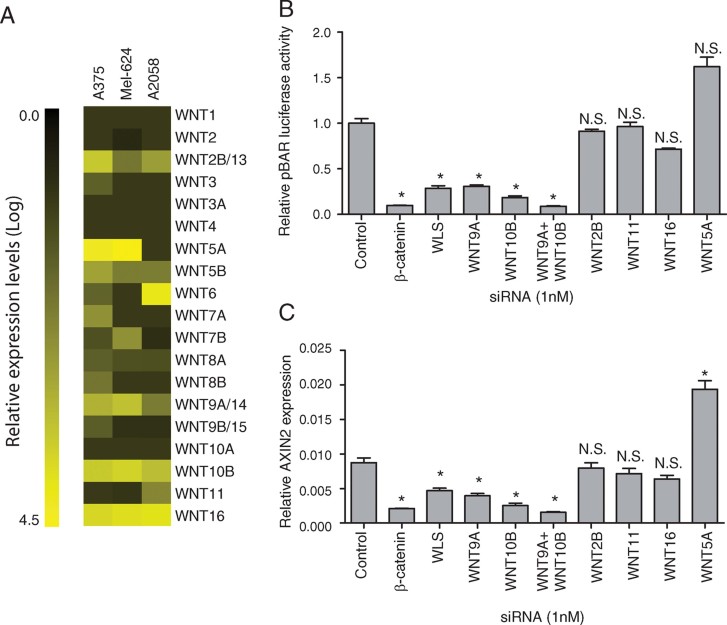
Expression of WNTs in melanoma Heat map representation of the relative expression of the WNT transcripts in A375, MEL624 and A2058 melanoma cells. The expression of WNT transcripts were normalized to *GAPDH* and given on a logarithmic scale. High expression is indicated in yellow and low expression in black.Analysis of WNT/β-catenin transcriptional reporter (BAR-luciferase) activity in A375 cells transfected with siRNAs targeting WNTs. BAR-luciferase values were normalized to a constitutively active renilla luciferase reporter to control for cell number, *p < 0.0001.Quantification of *AXIN2* mRNA in cells transfected with siRNAs targeting WNTs. *AXIN2* expression was normalized to *GAPDH*, *p < 0.005.

We next examined which WNT ligands activate endogenous WNT/β-catenin signalling observed in the melanoma cells used in this study. We first quantified the expression of all 19 endogenous *WNTs* in A375, MEL624 and A2058 cells (Fig 6A). Highly expressed *WNTs* included *WNT2B*, *WNT5A*, *WNT9A*, *WNT10B* and *WNT16*. In loss-of-function studies, we tested the effects of siRNAs targeting these WNT ligands in A375 cells. qPCR results confirm that siRNAs against individual WNT genes reduce endogenous transcripts compared to siRNAs against *WLS* or a non-silencing control siRNA (Supporting Information Fig S6). More importantly, WNT/β-catenin signalling as measured by BAR-luciferase is reduced by siRNAs targeting *WNT9A* or *WNT10B* as well as a pool of these siRNAs compared to siRNAs targeting *WNT5A*, *WNT11*, *WNT16* or a non-silencing control siRNA (*p* < 0.01, Fig 6B). These results are further supported by qPCR results demonstrating that siRNAs targeting *WNT9A* and *WNT10B* downregulate the expression of *AXIN2* (*p* < 0.0001 and *p* = 0.0014, respectively, Fig 6C). Together, these results strongly implicate WNT9A and WNT10B as candidates for regulating endogenous WNT/β-catenin signalling in these melanoma cells.

### Levels of *WLS* transcript are modulated by WNT/β-catenin in melanoma

Next, we explored possible factors contributing to the decreased levels of WLS observed in malignant melanomas (Fig 1). Previous studies indicate that overexpression of the *Drosophila WNT* homologue *wg* induces *wls* accumulation (Port et al, 2008) suggesting that *WLS* transcripts are either directly or indirectly modulated by WNT/β-catenin signalling. We then determined whether *WLS* is modulated by WNT/β-catenin signalling in melanoma cells. We monitored the activity of WNT/β-catenin signalling using a stably transduced β-catenin activated reporter (BAR) driving VENUS (Biechele et al, 2012; Coombs et al, 2010). When we analysed these reporter cells by flow cytometry, we find that a subset (5%) of A375 cells have relatively elevated endogenous WNT/β-catenin signalling based upon reporter-driven expression of VENUS. We compared the expression of *WLS* in VENUS-positive and VENUS-negative cells and found that *WLS* transcript levels are increased in the VENUS-positive cells (Supporting Information Fig S7A). These data indicate that *WLS* expression correlates with the activation of a WNT/β-catenin reporter.

We then tested if activating WNT signalling with exogenous WNT can induce *WLS* expression. Indeed, stimulating A375 cells with 10% WNT3A-conditioned media but not with control or WNT5A-conditioned media, increases the expression of both *WLS* (Supporting Information Fig S7B) and *AXIN2* (Supporting Information Fig S7C). These results suggest that *WLS* transcripts are induced by WNT3A signalling in melanoma. We next tested if β-catenin is required for WNT3A to induce *WLS* mRNA expression and found that siRNA against β-catenin inhibits the induction of *WLS* transcripts by WNT3A-conditioned media (Supporting Information Fig S7B). We also analysed expression profiles across a spectrum of melanocytes and additional melanoma cell lines and find that the expression of *WLS* and *AXIN2* are highly correlated (Spearman *R* = 0.8035, *p* = 0.0366, Supporting Information Fig S7D). Together, these data suggest that *WLS* expression may be regulated by the activation of WNT/β-catenin signalling in multiple melanoma cell lines.

## DISCUSSION

Our understanding of the role of WNT/β-catenin signalling in melanoma continues to evolve. In mouse models, activating WNT/β-catenin signalling using a melanocyte-specific, constitutively active β-catenin is by itself not sufficient to promote spontaneous melanoma tumourigenesis (Damsky et al, 2011; Delmas et al, 2007). However, constitutively active β-catenin promotes melanoma tumour initiation in the background of a mutant active Nras (Delmas et al, 2007). Knocking out β-catenin similarly inhibits melanoma tumourigenesis in mice carrying Braf and Pten mutations (Damsky et al, 2011). In contrast, our previous results using established murine and human melanoma cell lines indicate that activating WNT/ß-catenin signalling with WNT3A can inhibit the growth of melanoma cells *in vitro* and *in vivo* (Biechele et al, 2012; Chien et al, 2009b). Another recent study finds that either activating WNT/β-catenin signalling with a constitutively active β-catenin or knocking out β-catenin disrupts melanoblast proliferation in mouse embryos (Delmas et al, 2007; Luciani et al, 2011). Together, these results raise the possibility that a subtle balance in the activity of WNT/β-catenin signalling must be maintained during normal skin development and that either increasing or decreasing WNT/β-catenin signalling can permit melanoma tumourigenesis in a context-dependent manner.

Mutations known to result in β-catenin stabilization are detected in a small subset of human melanomas (∼7%; Hodis et al, 2012), which is in line with studies indicating that β-catenin can promote melanoma tumourigenesis in mouse models (Damsky et al, 2011; Delmas et al, 2007). Other studies suggest that reduced WNT/β-catenin signalling, scored as a reduction of nuclear or cytoplasmic β-catenin in melanoma patient samples, is associated with reduced patient survival (Bachmann, 2005; Chien et al, 2009b; Gould Rothberg et al, 2009). These data suggest that WNT/β-catenin signalling is either inhibited or lost in a subset of melanomas with poor clinical outcomes. In the present study, we pursued possible mechanisms that might lead to a down-regulation of WNT/β-catenin signalling in this subset of aggressive melanomas. Specifically, we investigate potential roles for a regulator of WNT secretion, WLS. We report that reducing WLS levels with shRNAs promotes spontaneous metastasis and growth in melanoma.

Strikingly, we find that *WLS* shRNA cells form spontaneous lung metastases at much greater frequency in comparison to control shRNA cells in mouse xenograft models. Yet, loss of WLS minimally affects cell migration and invasion *in vitro* suggesting that loss of WLS does not support a cell-intrinsic regulation of cell mobility. Instead, our proliferation studies support the hypothesis that loss of WLS may promote spontaneous metastasis by enhancing the survival and clonogenic growth of melanoma cells in the lungs. Our finding that WLS mediates melanoma cell proliferation through the regulation of WNT/β-catenin signalling not only strengthens the current model of WLS function in cancer cells, but also supports the idea that the activation of WNT/β-catenin signalling seen in a significant population of melanoma tumours is likely due to WNT ligand. Moreover, we find that *WLS* is itself regulated by WNT/β-catenin signalling, providing a means for feed-forward signalling that was previously unrecognized and suggesting that blocking WNT/β-catenin signalling can lead to a downregulation of *WLS* in melanoma cells.

Since WNT signalling pathways can be either up- or downregulated in different cancers, it is possible that regulating WLS specifically or WNT secretion more broadly could be a potential therapeutic strategy in a variety of cancers. Consistent with our results, a recent study reveals that WLS is overexpressed in malignant gliomas and that WLS promotes both WNT/β-catenin-dependent and -independent transcription in glioma xenografts (Augustin et al, 2011). In the present study, we observe that decreasing WLS expression in melanoma cells inhibits the WNT/β-catenin signalling pathway, promotes proliferation *in vitro* and enhances the formation of spontaneous lung metastases *in vivo*. Together, these results indicate that either inhibiting or activating WLS may serve as a novel therapeutic intervention in a cancer-type specific manner. In cancer contexts such as colorectal cancer where upregulated WNT/β-catenin is mediated by constitutively activating downstream mutations in *CTNNB1* or *APC*, therapeutic approaches to modulating WNT/β-catenin signalling have been somewhat limited. In cancers such as melanoma where ligand-driven activation of WNT/β-catenin is more common, strategies to manipulate WNT/β-catenin signalling are likely to be more feasible as a potential approach. Studies that clarify the temporal and spatial role of WNT/β-catenin in melanoma progression will further address if and how the targeting of this pathway could be useful for improving patient outcomes for this deadly disease. Since WLS has a G-protein-coupled receptor-like structure, which is often amenable to drug-targeting, we speculate that small molecule inhibitors or activators of WLS could be developed as novel cancer therapeutics.

## MATERIALS AND METHODS

### Cell culture

A375 (Catalog number CRL-1619, ATCC), A2058 and Mel 501 cells were cultured in DMEM containing 2% FBS. SK-Mel-2, SK-Mel-5 and SW480 cells were maintained in DMEM with 10% FBS. MEL624 cells were cultured in RPMI with 10% FBS. Melanoma cell cultures also contained 1% Penicillin–Streptomycin and 2.5 µg/ml Plasmocin (Invivogen, San Diego, CA, USA). HEMa-LP melanocytes were cultured in Medium 254 with PMA-Free Human Melanocyte Growth Supplement-2. Cell culture reagents were purchased from Life Technologies (Carlsbad, CA, USA) unless otherwise indicated.

### Cell transfection

Melanoma cells were transfected with 1 nM siRNA using 1 µl/ml Lipofectamine RNAiMAX, or 100 ng/ml constructs using 2 µl/ml Lipofectamine 2000 (Life Technologies). siRNAs against WNT5A, WNT2A, WNT9A, WNT10B, WNT11 and WNT16 were ordered from Life Technologies. Sequences of siRNAs are listed in the Supporting Information Materials and Methods.

### Generation of stable cell lines

Parental cell lines were infected with pGIPZ lentiviral constructs with non-silencing control, *GAPDH* or *WLS* shRNAs (line 1: V3LHS_343498 and line 2: V3LHS_343499), which were ordered from Open Biosystems (Thermo Scientific, Rockford, IL, USA). To clone GFP tagged WLS constructs, GFP tagged WLS or GFP was inserted into a second-generation lentiviral vector pHAGE (a gift from Balazs, AB and Mulligan, RC).

### Quantitative PCR

RNA samples were extracted with Trizol (Life Technologies) following manufacturer's instructions, and precipitated in the presence of 5 µg/ml glycogen (Life Technologies). One microgram of RNA was transcribed into cDNA using a Revertaid First Strand cDNA Synthesis Kit (Thermo Scientific), followed by quantification using SYBR green reagent and a LightCycler 480 system (Roche, Indianapolis, IN, USA). mRNA expression levels were normalized based on the expression of *GAPDH* or *RN18S1*. Primer sequences are listed in the Supporting Information Materials and Methods.

### Luciferase reporter assays

Three days after siRNA transfection, stable cell lines expressing the BAR-lucificerase reporter were directly lysed in the plates with Passive lysis buffer (Promega, Madison, WI, USA). Promega luciferase assay reagents were incubated with 10 µl cell lysate, and luciferase signal was measured using an Envision plate reader following manufacturer's instructions as previously described (Biechele et al, 2012).

### Live cell imaging

Migration of individual cells was monitored after plating them at low density (250 cells/cm^2^) on type I collagen coated plate (10 µg/cm^2^). Cells were pre-treated with 0.05 µM CHIR99021 (Cayman Chemical, Ann Arbor, MI, USA) or DMSO control 24 h before imaging and during the imaging analysis. More than 75 single cells were randomly chosen and monitored with Nikon TiE inverted widefield fluorescence microscope equipped with an Electron Multiplying Charge Coupled Device (EMCCD) camera and an environmental control chamber (37°C, 5% CO_2_). Pictures were taken in the GFP channel with minimal intensity and exposure time (20–50 ms) at 20 min intervals for at least 24 h. Pictures were then processed using NIS Elements software (Nikon Instruments Inc., Melville, NY, USA) to trace individual cell migration paths and to calculate velocity (path length/time, µm/s). Only cells traced for more than 18 h were included in the final dataset.

### Soft agar assay

Five thousand cells were embedded in 0.4% low-melting agar over a 0.8% base agar layer. In some experiments the 0.4% agar layer contained 10% control, WNT3A or WNT5A conditioned-media. Soft agar cultures were fed with 0.5 ml media twice a week. To visualize colonies, cultures were stained with 0.005% crystal violet (Sigma–Aldrich, St. Louis, MO, USA) overnight, and washed with PBS. Clusters with more than 20 cells were scored as colonies.

### Proliferation in two-dimensional culture

One-hundred melanoma cells were seeded in 96-well plates to monitor growth at low density. Media with reagents were changed twice a week. Small molecular compound XAV939 (Tocris Bioscience, Ellisville, MO, USA), CHIR99021 (Cayman Chemical) or 0.1% DMSO were included in the culture media. We captured images of individual wells in the GFP channel using an In Cell Analyzer 2000 high content imaging system (GE Healthcare). We then scored cell numbers using the In Cell Developer Toolbox (GE Healthcare). We first measured cell number after serial dilution of plated cells to verify the acquisition. Our statistical analysis reveals a significant correlation between seeded cell numbers and automated measurements (*R*^2^ = 0.994, *p* < 0.05, Supporting Information Fig S8).

### Western blot analysis

Cells were lysed with freshly prepared denaturing buffer containing 20 mM Tris–HCl (pH 6.5), 150 mM NaCl, 10 mM EDTA and 1% Triton X-100 (Sigma–Aldrich, St. Louis, MO, USA) with protease and phosphatase inhibitors (Roche), and followed by three freeze-thaw cycles in liquid nitrogen. Samples for Western blot analysis were combined with NuPAGE LDS sample buffer (Life Technologies) containing 0.05% β-mercaptoethanol, incubated at 95°C for 5 min and then loaded onto the NuPage 4%-12% *Bis*–*Tris* gel system (Life Technologies). The antibodies used in this study included: anti-WNT5A/B (1:1000, Cell Signaling Technology Danvers, MA, USA), anti-WNT10B (1:100, Aviva System Biology, San Diego, CA, USA), anti-GPR177 (1:3000, Abcam, Cambridge, MA, USA), anti-GFP (1:10,000, Abcam), anti-HSP90 (1:10,000, Abcam). The secondary antibodies were purchased from Jackson ImmunoResearch Laboratories (West Grove, PA, USA). To detect secreted proteins in media, equal numbers of cells were plated 18 h before adding low serum media DMEM or RPMI containing 0.5% FBS. Media was harvested after 48 h and concentrated using Amicon Ultra Centrifugal F with a10 kda cut-off (Millipore, Bedford, MA, USA). Intensity of Western blot signals was quantified with Adobe Photoshop (San Jose, CA, USA) and normalized with loading controls. Un-cropped immunoblot images are shown in Supporting Information Fig S9.

**The paper explained**

PROBLEM:

Melanomas cause the highest mortality among skin cancers. Recent evidence indicates that WNT/β-catenin signalling may be downregulated during melanoma progression. However, melanomas express high levels of endogenous WNT ligands. The mechanisms regulating the apparent insensitivity of melanomas to WNT ligand expression remain unclear.

RESULTS:

In this study, we observe a decrease in the WNT secretory receptor WLS levels across human melanoma cell lines and in patient tumours. Furthermore, we find that WLS may be functionally relevant in melanoma as reducing WLS levels enhances melanoma growth *in vitro* and the formation of spontaneous metastases *in vivo.* Conversely, overexpressing WLS can inhibit melanoma growth. As WLS is required for multiple WNT signalling pathways, we then determined whether WLS mediated changes in melanoma cell growth through β-catenin. Since small molecule inhibitors and activators of WNT/β-catenin signalling rescued the effects of WLS on melanoma cell proliferation, we conclude that WLS promotes WNT/β-catenin signalling in order to suppress melanoma cell growth.

IMPACT:

This study indicates that reduced WLS could be the cause of loss of WNT/β-catenin signalling in malignant melanoma. Our results implicate WLS as an important negative regulator of human melanoma cell growth and metastasis. Consequently, we predict that re-activating canonical WNT/β-catenin signalling by up-regulating WLS levels or blocking its negative regulators may serve a novel therapeutic strategy in melanoma.

### Xenografts

The indicated number of cells were suspended in a volume of 0.1 ml and injected into the flanks of NOD-SCID γ-immunocompromised mice (Jackson Laboratory, Maine, USA). The tumour volumes were calculated as described previously (Anastas et al, 2011). All animal studies were performed using Institutional Animal Care and Use Committee protocols as approved by a review board at the University of Washington.

### Immunohistochemistry of melanoma samples

Antibodies raised against the N-terminus of WLS with (sequence TEMAHERVPRKLK) were produced in guinea pigs (Pocono Rabbit Farm and Laboratory, Canadesis, PA, USA). Melanoma tumour samples were obtained from the University of Washington. Immunohistochemical staining of endogenous WLS was conducted at the Fred Hutchinson Cancer Research Center, Department of Experimental Histopathology. Establishment of WLS staining protocol was based on paraffin embedded sections of pelleted A375 cells expressing control or *WLS* shRNA (Supporting Information Fig S1A). Samples were deparaffinized and steamed in a pressure cooker for 20 min to facilitate antigen retrieval, followed by incubation in 4 µg/ml of affinity-purified serum against WLS for 60 min. To detect signal, samples were incubated in biotinylated goat anti-guinea pig secondary antibody (1:100, Vector, Burlingame, CA, USA) for 30 min and then with tertiary reagent Peroxidase conjugated with streptavidin for an additional 30 min (1:2000, Jackson ImmunoResearch). Additional sections were stained as described above using a guinea pig IgG (4 µg/ml) as an isotype control. Data supporting the specificity of WLS antibody are shown in Supporting Information Fig S1A, B and D. All studies involving human subjects were approved by an institutional review board under the supervision of the University of Washington Human Subjects Division (protocol #26161). The experiments performed conform to the principles set out in the WMA Declaration of Helsinki and the NIH Belmont Report.

### Statistical analyses

Shown numbers were obtained from at least three independent experiments. Results were plotted and analysed with GraphPad Prism (La Jolla, CA, USA). Error bars show SEM. Each condition was compared to control and determined by unpaired two-trailed *t*-tests with Welch's correction. Statistical differences in the proportion mice with lung metastases in xenograft experiments were determined using Fisher's exact tests (two-sided). Correlation studies were assumed nonparametric and analysed using a two-tailed Spearman's rank correlation coefficient. *p*-Values smaller than 0.05 were indicated as significant.

## Author contributions

PTY initiated the project and planned the experiments; RAT conducted xenograft studies. JMG performed qPCR on WNTs; AJC provided tumour samples; MMS and AJC scored histopathological staining on tumour samples; MMS and PTY provided images of histopathology; AKB provided critical discussions and comments; PTY wrote the paper; JNA, AJC and RTM provided critical discussion and edited the manuscript.

## For more information

Oncomine database:

https://www.oncomine.org/resource/login.html

Cited microarrays with accession number:

Pasmanik-Chor et al (2011) (E-GEOD-27980); Riker et al (2008) (E-GEOD-7553); Talantov et al (2005) (E-GEOD-3189); Valsesia et al (2011) (E-GEOD-22928); Winnepenninckx et al (2006) (E-TABM-1); Winnepenninckx et al (2007) (E-TABM-4).

## Supplementary material

Detailed facts of importance to specialist readers are published as ”Supporting Information”. Such documents are peer-reviewed, but not copy-edited or typeset. They are made available as submitted by the authors.
