# Supplementary material for: WLS inhibits melanoma cell proliferation through the β-catenin signalling pathway and induces spontaneous metastasis
Source: EMBO Mol Med. 2012 Nov 6;4(12):1294–307. doi: 10.1002/emmm.201201486 (PMC3531604; doi:10.1002/emmm.201201486)
Supplement: Supplementary file 1 [file emmm0004-1294-SD1.pdf]

EMBO Molecular Medicine

Manuscript EMM-2012-01486

WLS inhibits melanoma cell proliferation through the β-catenin signaling pathway and induces spontaneous metastasis

Pei-Tzu Yang, Jamie N. Anastas, Rachel A. Toroni, Michi M. Shinohara, Jamie M. Goodson, Anja K. Bosserhoff, Andy J. Chien, and Randall T. Moon

Corresponding author: Randall Moon, HHMI/University of Washington School of Medicine

Review timeline:

Submission date:	19 April 2012
Editorial Decision:	30 May 2012
Revision received:	28 August 2012
Editorial Decision:	17 September 2012
Revision received:	20 September 2012
Accepted:	24 September 2012

Transaction Report:

(Note: With the exception of the correction of typographical or spelling errors that could be a source of ambiguity, letters and reports are not edited. The original formatting of letters and referee reports may not be reflected in this compilation.)

1st Editorial Decision 30 May 2012

Thank you for the submission of your manuscript to EMBO Molecular Medicine. We have now heard back from the three referees whom we asked to evaluate your manuscript.

As you will see from the reports below, although all three reviewers find the study of interest, they also raise some issues that should be addressed in a major revision of the manuscript.

In particular Ref. 1 and 2 suggest strenghening the role of WLS in metastasis and recommend performing extra experiments. Ref. 2 and 3 are concerned about the introduction not properly introducing the topic and not citing the right references. I would therefore encourage you to modify this section of the manuscript accordingly. Finally, answering points 3 (Ref.1) and 3rd from last (Ref.3) should provide some additional mechanistic insight to the study.

Given these evaluations, I would like to give you the opportunity to revise your manuscript, with the understanding that the referee concerns must be fully addressed and that acceptance of the manuscript would entail a second round of review. Please note that it is EMBO Molecular Medicine policy to allow only a single round of revision and that, as acceptance or rejection of the manuscript will depend on another round of review, your responses should be as complete as possible.

Revised manuscripts should be submitted within three months of a request for revision; they will otherwise be treated as new submissions, except under exceptional circumstances in which a short extension is obtained from the editor. Also, the length of the revised manuscript may not exceed 60,000 characters (including spaces) and, including figures, the paper must ultimately fit onto

optimally ten pages of the journal. You may consider including any peripheral data (but not methods in their entirety) in the form of Supplementary information.

I look forward to seeing a revised form of your manuscript as soon as possible.

Yours sincerely,

Editor
EMBO Molecular Medicine

***** Reviewer's comments *****

Referee #1:

In the submitted manuscript, Yang and colleagues studied the role of Wnt secretion on proliferation and metastasis in melanoma. Previous reports that beta-catenin (canonical) Wnt signaling is reduced in melanoma, independent of the expression of Wnt ligands, indicating that changes in Wnt levels influence proliferation of melanoma cells in a context-dependent manner. However, the underlying mechanisms have not been fully understood. The regulation of Wnt signaling activity in melanoma has also been subject to quite some controversy, since - opposite to its role in e.g. colon cancer - a downregulation of beta-catenin-dependent Wnt activity has been linked to a reduced prognosis in patients.

In the present study, the authors found that a reduced expression of the Wnt secretion factor WLS in melanoma might be a possible explanation. Expression analysis of human melanoma biopsies revealed lower expression levels of the Wnt secretion factor WLS in malignant melanoma, indicating that WLS has a key function in controlling the amount of Wnt proteins independent of their expression levels. Previous studies have linked overexpression of WLS to glioma, which in this case leads to an activation of Wnt signaling activity.

This manuscript quite convincingly shows that WLS loss-of-function and gain-of-function influences Wnt-reporter activity and Wnt-target gene expression in melanoma cells. The Wnt ligand expression profile also confirmed that canonical Wnt signaling plays a major role in proliferation of melanoma cells. Tumor xenograft experiments revealed no significantly larger tumor after WLS silencing, however, interestingly reduced WLS expression in melanoma cells gave rise to more lung metastasis, and there is some indication (based on a retrospective study of public expression data) that this finding might hold also true in patients.

Overall, the study is well executed and interesting to the general readership of EMBO Moleculear Medicine. The finding that reduced beta-catenin signaling activity leads to an increase in melanoma is not particular novel, neither linking WLS to tumorigenesis (although here in the opposite direction), but it clarifies some of the conflicting data on the increase in Wnt expression and lower signaling activity which is controlled by WLS acting as a "gate-keeper". The most interesting and novel point is in my view linking WLS expression to metastasis, but this conclusion would need further experiments in support.

There are a number of major that should be addressed:

1. The finding that silencing of WLS leads to an increase in metastasis formation is probably the most interesting (and novel) finding of the manuscript. While the xenograft data appears to be solid (but the number of animals studies not very high), the conclusions about its relevance in patients only relies on a re-analysis of previously published microarray data (Wennepennickx et al., 2006) - which are also only marginally significant. I believe it is crucial that the authors further support this conclusion by their own analysis of tumor material using qPCR, IHC or WB. The authors should also state whether other public expression studies would not support this.

2. Migration assay with melanoma cells (A375, A2058) have been previously described and should be performed. This assay could give a better handle on why WLS silenced melanoma cells form metastasis.

3. WLS is known to be a transcriptional target of canonical Wnt signaling. Therefore the authors should analyze whether WLS expression is reduced in melanoma cells after beta-catenin silencing. High-non canonical signaling through WNT5A could lead to downregulation of WLS through inhibition of beta-catenin signaling. The authors should address this issue in more detail (e.g. expression of Wnt5a, monitoring of non-canonical Wnt signaling). These experiments might also provide a mechanistic link how high levels of WNT5A influence melanoma by downregulation of WLS.

Other points:

a. The flow of the presented data should be improved. The figures are not mentioned in chronological order and should be reordered. Re-organization of the text would be necessary to improve its clarity before publication.

b. Fig 1D-G are underwhelming. A quantitative analysis of IHC data should be provided. The authors should also demonstrate the specificity of the WLS antibody.

c. Overstatements should be avoided, e.g. "two stable cell lines" instead of "multiple stable cell lines"

d. Pictures of Fig 2E should be formatted similarly

e. Fig 3A (right panel) is not explained nor referenced in the text or Figure legend. Is this a rescue experiment with WNT5A?

f. Fig 3C. What is the effect of WNT5A in this assay?

g. The reviewer suggests to shift Fig 4 to the supplement.

h. Fig 4B is not correctly labeled. Full WB should be shown for readers to judge the specificity of the WLS antibody

i. When public data from e.g. oncomine is used, the authors should clarify how many studies support their conclusion to avoid selective interpretation of larger data sets

Referee #2:

In this paper the authors pursue their interest in the somewhat neglected topic of why Wnt signaling can promote cancer growth in some contexts and inhibit it in others. Melanomas offer an excellent context to study the Wnt pathway's inhibitory role in cancers. Additionally, melanomas show Wnt expression, and paradoxically low nuclear beta-catenin levels. How can this be? The authors show that a possible explanation is that the Wnts aren't secreted, because WLS is repressed. This is a satisfying answer and the data support it nicely.

One concern about the manuscript is that this reviewer failed to realize how nice this problem was until the end of the manuscript. The abstract and introduction should set the problem up more explicitly. As it stands, the introduction is difficult to read and should be rewritten.

The authors set up the medical relevance of the findings nicely in the conclusions. However, their best experiment, the in vivo quantification of lung metastases in mice injected with siRNA WLS A375 cells, offers an excellent way to test their hypothesis. A compound activating the Wnt pathway (GSK3 inhibitor for example) should be able to reduce the number of metastases, or reduce their size/number etc.

Overall, this is a nice paper proposing an interesting mechanism to explain some paradoxical findings.

Referee #3:

Yang et al. have used melanoma cell lines to evaluate the importance of WLS in melanoma proliferation and metastasis. WLS certainly appears to be an important regulator of these processes and its overall role in melanomagenesis is of interest. However, the principal role of WLS in oncological aspects remains elusive and the underlying molecular mechanism remains unclear. The structure and function of WLS are not considered and this article contains many imprecisions and errors that should be corrected.

Firstly, Mitf is not a homeobox gene (introduction: "the key homeobox gene micropthalmia transcription factor (MITF)"). It is actually a basic helix-loop-helix leucine zipper transcription factor. This should be corrected.

The sentence "However, the role of WNT/ -catenin signaling in melanoma progression remains unresolved since activation of WNT/ -catenin signaling promotes melanoma growth in mouse models of tumorigenesis driven by Nras (Delmas et al., 2007), while loss of WNT/ -catenin prevents mortality in a mouse model of Pten-deficient melanoma (Damsky et al., 2011)" is inappropriate and misleading, for various reasons:

1) These two articles (Damsky and Delmas) deal principally with the initiation of melanoma formation rather than with melanoma progression.
2) Damsky et al. (2011) used various mouse models. No melanoma formation is observed in mice in the absence of PTEN; the presence of an activated form of BRAF (BRAFV600E) is required.
3) The articles by Delmas et al. and Damsky et al. are not contradictory. Delmas et al. reported that melanocytes produce activated forms of NRAS and -catenin. Damsky et al. reported that melanocytes produce activated forms of BRAF, PTEN and -catenin. These different combinations of mutations lead to melanoma.
4) However, it is entirely true that melanoma formation is inhibited in the absence of bcat. The authors should refer to the article by Luciani (2011) evaluating the consequences of a lack of bcat for proliferation during lineage establishment. This would clearly provide a better introduction to this work.

Finally, although we agree with the authors that "the role of WNT/ -catenin signalling in melanoma progression remains unresolved" we do not agree with their reasons. The authors should rewrite the introduction, being more cautious in their assertions and providing useful and appropriate information.

Figure 1AB. Based on histology, what is the ratio of melanocyte/melanoma vs other cells in the skin, naevi and malignant melanoma?

Figure 1C. Were different culture media used for melanocytes and melanoma cells? If yes, what effect did the use of different culture media have on WLS expression?

Figure 1D. The authors should indicate the precise region of WLS used to generate the antibody. This experiment is certainly important and reveals that WLS is present mostly in keratinocytes and non-melanocyte cells.

The authors emphasise "the hypothesis that WLS is reduced with melanoma progression", which is entirely possible. However, they should not forget another important hypothesis: that WLS is less strongly expressed in melanocytes/melanoma cells than in other cells. This should be highlighted in the results section.

The scale bars are clearly incorrect in Fig. 1D-G'. This size cannot possibly correspond to 1 μm. Higher magnification images should be acquired and shown.

The sections should be double-stained with an appropriate marker of melanocytes.

The images are not of a sufficiently high quality for an adequate evaluation of the smooth muscles. The authors should either present higher-quality images or, even better, carry out appropriate

double-immunostaining.

I am surprised to find that Fig. 4A is cited just after Fig. 1G. The same is also true of Fig 4B, it is called to early.

Which A375 cell line was used in this study? At least two A375 subclones exist, and they have very different characteristics. Did the authors really inject 500 and 1000 cells into each mouse?

GFP detection is poor in Fig. 2E. This should not be too difficult to fix.

The authors should evaluate the major components of the Wnt/b-catenin signalling pathway in A375 + WLS Sh lung metastasis. Is WLS normally produced in the lung? What consequence does WLS Sh have in a melanoma cell line with a -catenin mutation that does not normally form lung metastases? The reverse experiment could also be performed.

The authors should pay attention to basic errors, such as the misspelling of "crystal" as "chrystal", for example. They should also try to express themselves more concisely in the text. For instance, the following is from the legend of figure 3A. "A375 cells were embedded at single cell density in 0.4% soft agar. Colonies were stained with crystal violet solution. Pictures were taken by 10X object (left) and colony numbers were quantified (right). Scale bars are 1 mm. Crystal violet staining of colonies from cells stably expressing GFP or WLS-GFP after 3 weeks of growth in soft agar". This, and similarly complex phrases elsewhere in the text, should be simplified.

In Figure 3D, the authors do not comment on the effects of GAPDH shRNA on the growth or production/localisation of WNT5A/B proteins in A375 melanoma cell lines. They should comment on these aspects.

1st Revision - Authors' Response 28 August 2012

Response to editor's comment:

Comment 1: In particular Ref. 1 and 2 suggest strengthening the role of WLS in metastasis and recommend performing extra experiments.

To assess the role of WLS in metastasis, we followed the Reviewers' suggestion and performed migration and invasion assays *in vitro*. We found that *WLS* shRNAs enhanced the migration of individual cells, however the effect was not strong (Fig S5C of Supporting Information). Furthermore, we did not observe any differences in wound-healing assays or in spheroid assays (Fig S5A and B of Supporting Information). These results suggest that *WLS* shRNA may promote metastasis by enhancing cell proliferation in low density (Fig 3) or via other, as yet undiscovered, mechanisms.

We agree that activating WNT signalling in cells expressing *WLS* shRNAs in xenograft studies would provide significant mechanistic insight regarding the role of WNT signalling in metastasis. Unfortunately, activating WNT/β-catenin *in vivo* is technically challenging and will produce data that is difficult to interpret. GSK3β inhibitors can be used to activate WNT signalling downstream of WNT ligands, yet these inhibitors are not specific and are not ideal for extended use *in vivo.* More importantly, systemic treatment with GSK3β inhibitors will activate WNT signalling both in the xenografted melanoma cells as well as in the host tissue, making results of these types of experiments difficult to interpret. We have included a more detailed explanation of the technical and experimental challenges involved in the use of GSK3β inhibitors in our response to the reviewers' comments (Page 6 and 7).

© EMBO

As an alternative approach, we also attempted to activate WNT signalling in the *WLS* shRNA cells by expressing a stabilized form of β-catenin. Surprisingly, β-catenin reduced cell viability making it difficult to maintain stable cell lines and to acquire sufficient cells to conduct the xenograft experiments (see Reviewer Fig 6). Finally, we infected Mel 501 human melanoma cells, which harbour point-mutated β-catenin, with either *WLS* or control shRNAs. We find that *WLS* shRNAs reduce the growth of Mel 501 cells (Reviewer Fig 7). Since we obtained opposite results by transducing Mel 501 cells with *WLS* shRNAs in comparison to A375, MEL624, and A2058 cells where *WLS* shRNAs enhance growth, we were discouraged from proceeding with the xenografts experiments. The reasons for the conflicting result obtained in Mel 501 cells are not yet clear, but could involve cell-line specific differences in oncogenes or tumour suppressors, or perhaps differences in WNT expression profiles.

In summary, we agree that activating β-catenin signalling downstream of WNT ligands could significantly improve our understanding of the role of WLS in melanoma metastasis. Unfortunately, technical challenges and the cell line- specific differences that we describe above suggest that these studies will require a significant amount of work to address appropriately. Given the length limits of a typical article in EMBO Molecular Medicine, we believe that these experiments are better suited for an independent project.

Comment 2: Ref. 2 and 3 are concerned about the introduction not properly introducing the topic and not citing the right references. I would therefore encourage you to modify this section of the manuscript accordingly.

We simplified the introduction and focused on citing references from studies analysing patient melanoma samples, which are more relevant to the human melanoma cell lines that we used in this study. In the new version of the introduction we emphasize the main focus of the paper, which is to investigate a potential role for WNT secretion in melanoma. We have also moved our discussion of some of the unresolved issues related to the role of WNT signalling in melanoma and the corresponding citations to the discussion section.

Comment 3: Finally, answering points 3 (Ref.1) and 3rd from last (Ref.3) should provide some additional mechanistic insight to the study.

In response to Referee 1 point 3, we conducted additional experiments to determine whether *WLS* is a target of either the WNT/β-catenin pathway or a target of WNT5A (Supporting Figure S2). Specifically, we find that WNT3A up-regulates *WLS* expression in a β-catenin-dependent manner. We also demonstrate that WNT5A does not regulate the expression of *WLS,* suggesting *WLS* expression is not regulated by non-canonical WNT signalling in this context. We have also shown that reducing *WLS* with shRNAs inhibits WNT5A secretion without altering *WNT5A* transcript expression (Fig S4 of Supporting Information), suggesting that non-canonical WNT signalling is down-regulated in cells expressing *WLS.* We further supported this finding by determining the level of phosphorylated PKC, a target of WNT5A-dependent signalling in melanoma (Weeraratna et al 2002). Indeed, WLS loss of function moderately decreases PKC phosphorylation (Reviewers Fig 1).

Given that there are 19 different WNTs, more than a dozen different WNT receptors, and numerous kinases, ubiquitin ligases, transcription factors and scaffolding proteins known to be involved in WNT signalling (See " The WNT homepage" http://www.stanford.edu/group/nusselab/cgi-bin/wnt/), we believe that Referee 3's request for an analysis of the components of WNT signalling in lung metastases is better suited for a follow-up study.

Comment 4: In support of Referee's 1 point h, we encourage the publication of source data, particularly for electrophoretic gels and blots, with the aim of making primary data more accessible and transparent to the reader. Would you be willing to provide a single PDF file comprising the original, uncropped and unprocessed scans of all or key gels used in the figures? These should be

labelled with the appropriate figure/panel number, and should have molecular weight markers; further annotation could be useful but is not essential. This PDF could be published online with the article as a supplementary "Source Data" file. If you have any questions regarding this just contact me.

We include all of the unprocessed immunoblot images in Fig S9 of Supporting Information.

Point-by-Point-Response to Referees:

Response to Referee #1:

Referee #1 Comment 1: The finding that silencing of WLS leads to an increase in metastasis formation is probably the most interesting (and novel) finding of the manuscript. While the xenograft data appears to be solid (but the number of animals studies not very high), the conclusions about its relevance in patients only relies on a re-analysis of previously published microarray data (Wennepennickx et al., 2006) - which are also only marginally significant. I believe it is crucial that the authors further support this conclusion by their own analysis of tumour material using qPCR, IHC or WB. The authors should also state whether other public expression studies would not support this.

Answer 1: We thank the reviewer for his/her insightful suggestions throughout the manuscripts, as addressing these issues has greatly enhanced the manuscript. We agree that analysis of WLS expression in additional patient samples might provide evidence supporting a role for WLS in suppressing melanoma growth and metastasis, which would be consistent with our xenograft and *in vitro* studies. We therefore included a more comprehensive analysis of *WLS* expression described below. As an independent confirmation we have extended our analysis of WLS protein expression by IHC. Regarding the IHC data, we have included two metastatic tumours from melanoma patients (Figure 1I-K, and Table S1 of Supporting Information), and find that WLS staining is absent in the dermal cells of both tumours. While we refrain from making conclusions based on two tumours (and no further tumour tissue is available), these results are nevertheless *consistent* with our analysis of microarray studies. Taken together the data support our conclusion that WLS levels may be reduced in melanoma.

We also attempted to analyse the expression of *WLS* in datasets including expression profiles of metastatic melanomas compared to paired primary tumours. Unfortunately reference expression values are not available in the publically available datasets that we found, making statistical analysis difficult.

Summary of WLS expression in microarray studies by comparing matched primary and metastatic tumours. Fold change was calculated dividing the average expression of WLS in metastatic tumour samples by the average expression of WLS in primary tumours from the same patient. The microarrays dataset did not contain the reference values from primary melanomas (indicated by ¶), which limits statistical analysis.

Referee #1 Comment 2: Migration assay with melanoma cells (A375, A2058) have been previously described and should be performed. This assay could give a better handle on why WLS silenced melanoma cells form metastasis.

Answer 2: We investigated the effect of WLS shRNA in terms of melanoma cell motility by tracking the migration of individual cells in live cell imaging experiments and by performing wound-healing assays. We did not observe any differences in the migration rate of A375 cells expressing different shRNAs in scratch assays (Fig S5). However, when we tracked the migration of individual A375 cells plated at low density (250 cells/cm^2), we find that A375 cells transduced with WLS shRNAs migrate faster than cells transduced with control shRNA (n>75, P<0.001) (Fig S6). Although the increased migration of *WLS* shRNA cells in comparison to control cells was statistically significant, the relative increase in migration rate is very small (18%±12%). Since we did not see any effect of WLS loss of function in wound healing assays and the effect of *WLS*

© EMBO

shRNA on individual cell migration was slight, we concluded that loss of WLS does not greatly enhance or inhibit cell migration *in vitro.*

Referee #1 Question 3: WLS is known to be a transcriptional target of canonical Wnt signalling. Therefore the authors should analyse whether WLS expression is reduced in melanoma cells after beta-catenin silencing.

Answer 3: We have performed additional experiments as requested and find that *WLS* can be up-regulated by activating WNT3A/β-catenin-dependent signalling in melanoma cells. We used a reporter cell line harbouring a β-catenin responsive DNA element driving the expression of GFP to identify melanoma cells with endogenously activated WNT/β-catenin signalling (Biechele and Moon, Methods Mol Bio, 2008). We then isolated GFP positive cells with high WNT/ β-catenin signalling by fluorescence-activated cell sorting (FACs) and find that *WLS* expression is higher in GFP positive cells compared to GFP negative cells (Fig S8A). These results suggest that WLS expression correlates with WNT/β-catenin pathway activation. We then asked whether β-catenin is required to maintain WLS transcript levels in melanoma cells. When we transfected melanoma cells with *β-catenin* siRNA we observed no change in *WLS* expression (Fig S8), indicating that endogenous WNT/β-catenin signalling does not regulate *WLS* transcript levels.

Next, we asked whether treatment with exogenous WNTs regulate WLS expression. We observed an increase in *WLS* expression by approximately 3 fold following treatment with WNT3A conditioned-media (Fig S8B). Finally we asked whether WNT3A promotes WLS expression via β-catenin signalling and find that *β-catenin* siRNA blocks the induction of *WLS* induced by WNT3A, suggesting that a WNT3A/β-catenin pathway can induce *WLS* expression. We agree that the expression of *WLS* may be regulated by many different factors including both β-catenin-dependent and –independent WNT signalling. However, our paper focuses on understanding the functional relevance of WLS in melanoma rather than on the regulation of *WLS* expression. We would argue that a more extensive analysis of the determinants of *WLS* expression is better suited for a follow-up study.

Referee #1 Question 4: High-non canonical signalling through WNT5A could lead to down regulation of WLS through inhibition of beta-catenin signalling. The authors should address this issue in more detail (e.g. expression of Wnt5a, monitoring of non-canonical Wnt signalling). These experiments might also provide a mechanistic link how high levels of WNT5A influence melanoma by down regulation of WLS.

Answer 4: We did not observe any significant changes in either *WLS* or *AXIN2* expression following treatment with WNT5A conditioned media (Fig S8B and S8C), indicating that non-canonical WNT signalling through WNT5A does not inhibit β-catenin or *WLS* transcripts in this context.

We have also analysed the function of WLS in terms of WNT5A dependent signalling. We found that *WLS* shRNAs inhibited endogenous WNT5A secretion without changing *WNT5A* transcripts (Fig S2A and S2B). These results indicate that WLS can regulate both β-catenin-dependent and -independent WNT signalling in melanoma. In our paper we have focused on β-catenin-dependent signalling based on the results of our rescue experiments (Figures 4). Specifically, we show that blocking β-catenin-dependent signalling with XAV939 can rescue the effects of *WLS* gain of function (Figure 5), and activating β-catenin signalling with a GSK3β inhibitor can rescue the effects of *WLS* loss of function (Figure 4). We are pleased that the reviewer has raised the question of non-canonical Wnt signalling in melanoma and we wish to note that we are preparing an even longer manuscript on that subject.

Referee #1 Comment 5: The flow of the presented data should be improved. The figures are not mentioned in chronological order and should be reordered. Re-organization of the text would be necessary to improve its clarity before publication.

Answer 5: We have changed the figures accordingly.

Referee #1 Comment 6: Fig 1D-G are underwhelming. A quantitative analysis of IHC data should be provided. The authors should also demonstrate the specificity of the WLS antibody.

Answer 6: A board certified pathologist has scored our WLS IHC expression data and we have added these results to the Table S1 of Supporting Information. To demonstrate the specificity of WLS antibody, we performed IHC on paraffin embedded A375 cells expressing either control or *WLS* shRNA. We find that *WLS* shRNA reduced the IHC signal intensity observed with our WLS antibody, which suggests that the signal is specific (Fig S1A).

Referee #1 Comment 7: Overstatements should be avoided, e.g. "two stable cell lines" instead of "multiple stable cell lines"

Answer 7: We have corrected these phrases. However, we had initially generated multiple stable cell lines by transducing melanoma cells with six different WLS shRNAs. We validated knockdown of WLS with these shRNAs by western blotting. We then selected lines transduced with two of the six independent shRNAs (*WLS* shRNA 343498 and 343499) for further use in our study.

Referee #1 Comment 8. Pictures of Fig 2E should be formatted similarly

Answer 8: We apologize for the inconsistency of picture shapes. To orientate the lungs in the same direction, we had to rotate the pictures. Therefore, some of the pictures are missing corners. No specific features have been enhanced, obscured, removed, or introduced due to these image manipulations. Please find un-edited versions of the photographs included in Figure 2E.

Referee #1 Comment 9. Fig 3A (right panel) is not explained nor referenced in the text or Figure legend. Is this a rescue experiment with WNT5A?

Answer 9: The right panel of Fig 3A is the quantification of colony numbers in the soft agar assay. We have separated Fig 3A into two panels for clarification. These panels are now in Fig 4E (pictures) and 4F (quantification) in the revised version.

Referee #1 Comment 10. Fig 3C. What is the effect of WNT5A in this assay?

Answer 10: WNT5A-conditioned media does not have any effect on colony formation in soft agar and did not rescue the effect of WLS shRNAs (Fig 4E and 4F).

Referee #1 Comment 11. The reviewer suggests moving Fig 4 to the supplement.

Answer 11: We moved Fig 4 to Fig S2 of Supporting Information as suggested.

Referee #1 Comment 12. Fig 4B is not correctly labelled. Full WB should be shown for readers to judge the specificity of the WLS antibody

Answer 12: We have included un-cropped scans of our western blots in the Supporting Information as requested (Fig S9).

Referee #1 Comment 13. When public data from e.g. oncomine is used, the authors should clarify how many studies support their conclusion to avoid selective interpretation of larger data sets

Answer 13: We analysed *WLS* expression in a total of three microarray studies comparing human skin with melanoma tumour samples. Two out of three studies support that *WLS* is decreased in melanomas. We also analyse total two microarrays comparing benign nevi with melanomas, and both of them support the same conclusion

Response to Referee #2:

Referee #2 Comment 1: One concern about the manuscript is that this reviewer failed to realize how nice this problem was until the end of the manuscript. The abstract and introduction should set the problem up more explicitly. As it stands, the introduction is difficult to read and should be rewritten.

Answer 1: We have revised our introduction to clarify our main points.

Referee #2 Comment 2: The authors set up the medical relevance of the findings nicely in the conclusions. However, their best experiment, the in vivo quantification of lung metastases in mice injected with siRNA WLS A375 cells, offers an excellent way to test their hypothesis. A compound activating the Wnt pathway (GSK3 inhibitor for example) should be able to reduce the number of metastases, or reduce their size/number etc.

Answer 2: We agree that activating WNT signalling downstream of WNT ligands would provide significant mechanistic insight regarding the role of WNT secretion in melanoma metastasis. Unfortunately pharmacological activation of WNT/CTNNB1 *in vivo* poses both technical and experimental challenges. First of all, the specific activation of the WNT/CTNNB1 pathway is difficult due to the lack of specific drugs that are appropriate for use *in vivo* (Pan et al., Neuropsychopharmacology, 2011, and our unpublished results). Many GSK3β inhibitors commonly used *in vivo* such as, LiCl and BIO ((2'Z,3'E)-6-bromoindirubin-3'–oxime) lack specificity and also inhibit the activity of a variety of other kinases (Pan et al., 2011). Although the drug CHIR99021 is a potent and specific inhibitor of GSK3β *in vivo* and *in vitro* (Pan et al., 2011), the half-life of CHIR99021 is very short (0.87 hour) in mice (Pan et al., 2011). Indeed, our group could only activate a WNT reporter (BAT-GAL) with a high dose (12.5 mg/kg) of CHIR99021 administered by intraperitoneal injection everyday. Daily injections over a period of 40+ days needed for the metastasis assay will likely cause significant stress in the mice used in the study and could confound the results.

Second, treatments with GSK3β inhibitors will not only activate WNT signalling in the transplanted melanoma cells, but will also alter the activity of signal transduction pathways, metabolism, and other aspects of host tissue physiology. Previous studies implicate WNT and GSK3β-dependent signalling in many aspects of skin biology. WNT signalling regulates the behaviour of hair follicle stem cells during regeneration (Greco et al, Cell Stem Cell, 2009), and sustained activation of β-catenin, or inhibition of GSK3β leads to skin fibrosis (Hamburg et al, JID, 2012; Bergmann et al, Rheum Dis. 2011). GSK3β also coordinates cell polarity and regulates the migration of skin progenitor cells (Wu et al, Cell, 2011). Finally, GSK3β inhibitors have also been recently shown to inhibit melanoma cells from adhering to skin fibroblasts, and to inhibit the transendothelial migration of melanoma cells (John et al, JID, 2012). These studies suggest that long-term treatment with GSK3β inhibitors will significantly alter the behaviour of the mouse host tissue in the skin and will render the results of any studies involving treatment with GSK3β inhibitors uninterpretable.

Additional studies suggest that GSK3β inhibitors also alter the microenvironment in the host lung tissue. In the lung, WNT signalling plays an important role in the regulation of epithelial stem cell numbers and in the regeneration of bronchioavelolar tissue in response to injury (Zhang Y et al, Nature Genetics, 2008). WNT signalling also regulates the proliferation and differentiation of mesenchymal stem cells in the lung including, smooth muscle cell progenitors (Cohen et al, JCI, 2009; Wang et al, MCB, 2005). Together, these studies suggest that long-term treatment with GSK3β inhibitors will alter the tumour microenvironment both in the skin at the primary site of injection and in the lungs where we observe metastatic tumours.

Referee#2 comment 3: Overall, this is a nice paper proposing an interesting mechanism to explain some paradoxical findings.

Answer 3: Thank you.

Response to Referee #3:

Referee #3 Comment 1: Firstly, Mitf is not a homeobox gene (introduction: "the key homeobox gene micropthalmia transcription factor (MITF)"). It is actually a basic helix-loop-helix leucine zipper transcription factor. This should be corrected.

Answer 1: We thank the reviewer for comprehensive suggestions and comments on the literature citing, as addressing these issues greatly enhance the clarity of manuscript. We have corrected this error in our revised introduction.

Referee #3 Comment 2: The sentence "However, the role of WNT/β-catenin signalling in melanoma progression remains unresolved since activation of WNT/β-catenin signalling promotes melanoma growth in mouse models of tumorigenesis driven by Nras (Delmas et al., 2007), while loss of WNT/β-catenin prevents mortality in a mouse model of Pten-deficient melanoma (Damsky et al., 2011)" is inappropriate and misleading, for various reasons:

1. *These two articles (Damsky and Delmas) deal principally with the initiation of melanoma formation rather than with melanoma progression.*

2. *Damsky et al. (2011) used various mouse models. No melanoma formation is observed in mice in the absence of PTEN; the presence of an activated form of BRAF (BRAFV600E) is required.*

3. *The articles by Delmas et al. and Damsky et al. are not contradictory. Delmas et al. reported that melanocytes produce activated forms of NRAS and β-catenin. Damsky et al. reported that melanocytes produce activated forms of BRAF, PTEN and β-catenin. These different combinations of mutations lead to melanoma.*

Answer 2: We agree that both the studies from both Damsky et al and Delmas et al suggest that WNT/β-catenin signalling promotes melanoma development in mouse models. We had written that, "the role of WNT/β-catenin signalling in melanoma remains unresolved" in reference to the two preceding sentences:

> "…previous studies report an unexpected correlation between elevated ß-catenin in malignant melanoma and increased patient survival, and conversely, a correlation between lower levels of ß-catenin and reduced survival (Arozarena et al, 2011; Chien et al, 2009b; Kageshita et al, 2001; Kuphal & Bosserhoff, 2011; Maelandsmo et al, 2003). Our previous studies also reveal that activating WNT/β-catenin signalling by over-expressing WNT3A in mouse or human melanoma cells decreases cell proliferation and tumorigenesis in xenograft assays (Biechele et al, 2012; Chien et al, 2009b)."

Hence, the role of WNT/β-catenin signalling in melanoma remains unresolved due to conflicting results obtained using different model systems. We have moved this discussion of potential roles for WNT/β-catenin in various models of melanoma to the discussion section and have revised the text as follows:

"Our understanding of the role of WNT/β-catenin signalling in melanoma continues to evolve. In mouse models, activating WNT/β-catenin signalling using a melanocyte-specific, constitutively active β-catenin is by itself not sufficient to promote spontaneous melanoma tumorigenesis (Damsky *et al*, 2011; Delmas *et al*, 2007). However, constitutively active β-catenin promotes melanoma tumour initiation in the background of a mutant active Nras (Delmas *et al*, 2007). Knocking-out β-catenin similarly inhibits melanoma tumorigenesis in mice carrying Braf and Pten mutations (Damsky *et al*, 2011). In contrast, our previous results using established murine and human melanoma cell lines indicate that activating WNT/ß-catenin signalling with WNT3A can inhibit the growth of melanoma cells *in vitro* and *in vivo* (Chien *et al*, 2009b; Biechele *et al*, 2012). Another recent study finds that either activating WNT/β-catenin signalling with a constitutively active β-catenin or knocking out β-catenin disrupts melanoblast proliferation in mouse embryos (Delmas *et al*, 2007; Luciani *et al*, 2011). Together, these results raise the possibility that a subtle balance in the activity of WNT/β-catenin signalling must be maintained during normal skin development and that either increasing or decreasing WNT/β-catenin signalling can permit melanoma tumorigenesis in a context-dependent manner."

Referee #3 Comment 3: However, it is entirely true that melanoma formation is inhibited in the absence of β-catenin. The authors should refer to the article by Luciani (2011) evaluating the consequences of a lack of bcat for proliferation during lineage establishment. This would clearly provide a better introduction to this work.

Answer 3: We have revised the text of our introduction to include the results of this important study summarized in the following statement:

"Another recent study finds that either activating WNT/β-catenin signalling with a constitutively active β-catenin or knocking out β-catenin disrupts melanoblast proliferation in mouse embryos (Delmas *et al*, 2007; Luciani *et al*, 2011)"

Referee #3 Comment 4: Finally, although we agree with the authors that "the role of WNT/β-catenin signalling in melanoma progression remains unresolved" we do not agree with their reasons. The authors should rewrite the introduction, being more cautious in their assertions and providing useful and appropriate information.

Answer 4: We have rewritten the introduction to clarify our rationale for studying WNT secretion in the context of melanoma. Specifically, we have emphasized evidence from patient tumours and experiments involving human melanoma cell lines, which we believe are most relevant to our current study. Using patient tumour samples, which are more relevant to the human melanoma cell lines used in this study.

Referee #3 Comment 5: Figure 1AB. Based on histology, what is the ratio of melanocyte/melanoma vs other cells in the skin, naevi and malignant melanoma?

Answer 5: According to the published articles, only samples comprised of more than 95% tumour cells were analysed in the studies by Haqq et al., and Riker et al., Talantov and colleagues did not state the percentage of melanoma cells in the samples, but they mentioned more than 50% melanocyte content with no mixed histology in the melanoma and benign nevi analysed in their study.

Referee #3 Comment 6: Figure 1C. Were different culture media used for melanocytes and melanoma cells? If yes, what effect did the use of different culture media have on WLS expression?

Answer 6: Yes, we cultured melanocytes (HEMa-LP) in Medium 254 following the instructions from the vendor (Life Technologies), and A375 cells in DMEM (Biechele et al, Science Signaling, 2012) as described in Materials and Methods. We have been unsuccessful at culturing melanocytes in other media, but were able to maintain A375 cells in Medium 254 and Opti-MEM. We observed no statistically significant changes in *WLS* expression in A375 cells in response to culturing them in different media, but were able to reduce *WLS* expression with shRNA in different media. The results of quantitative PCR are included below.

Referee #3 Comment 7: Figure 1D. The authors should indicate the precise region of WLS used to generate the antibody. This experiment is certainly important and reveals that WLS is present mostly in keratinocytes and non-melanocyte cells.

Answer 7: We had included the sequence of synthetic peptide used to generate the antibody used in IHC staining in the Materials and Methods section of our paper, TEMAHERVPRKLK. This sequence corresponds to the extracellular domain of WLS.

Referee #3 Comment 8: The authors emphasise "the hypothesis that WLS is reduced with melanoma progression", which is entirely possible. However, they should not forget another important hypothesis: that WLS is less strongly expressed in melanocytes/melanoma cells than in other cells. This should be highlighted in the results section.

Answer 8: We stained serial sections of normal human skin with the melanocyte marker S100 and find that WLS expression overlaps with this maker suggesting that the levels of WLS are indeed higher in melanocytes compared to other cell types in normal human skin. We have edited our results section as follows:

"We also observe that the regions staining positively for WLS overlap with the melanocyte marker S100 in adjacent serial sections (Fig 1F, indicated by arrow), suggesting that WLS protein is expressed in melanocytes, but is either weakly expressed, or absent in other cell types present in normal human skin."

Referee #3 Comment 9: The scale bars are clearly incorrect in Fig. 1D-G'. This size cannot possibly correspond to 1 µm. Higher magnification images should be acquired and shown.

Answer 9: We have corrected the scale bars in the revised figure. We have also added images taken at higher magnification as requested (Supporting Information Fig S1C).

Referee #3 Comment 10: The sections should be double-stained with an appropriate marker of melanocytes.

Answer 10: We have now included IHC staining of melanocyte marker S100 and find that WLS signal overlaps with S100 in the adjacent serial section (Fig 1E and 1F).

Referee #3 Comment 11: The images are not of a sufficiently high quality for an adequate evaluation of the smooth muscles. The authors should either present higher-quality images or, even better, carry out appropriate double-immunostaining.

Answer 11: We have now included IHC staining of smooth muscle marker SMA (Alpha smooth muscle actin) and find that WLS staining overlaps with this marker in the adjacent serial section (Fig S1C).

Referee #3 Comment 12: I am surprised to find that Fig. 4A is cited just after Fig. 1G. The same is also true of Fig 4B, it is called to early.

Answer 12: We have rearranged the figures and result section to keep figures in chronological order as much as possible.

Referee #3 Comment 13: Which A375 cell line was used in this study? At least two A375 subclones exist, and they have very different characteristics.

Answer 13: We used A-375[A375] cells from ATCC, the order number was specified in the Materials and Methods.

Referee #3 Comment 14: Did the authors really inject 500 and 1000 cells into each mouse?

Answer 14: Yes, we injected 500 or 1000 cells subuctaneously in each mouse.

Referee #3 Comment 15: GFP detection is poor in Fig. 2E. This should not be too difficult to fix.

Answer 15: We have adjusted the brightness/contrast to better show GFP expression in these photographs (Fig 2E).

Referee #3 Comment 16: The authors should evaluate the major components of the Wnt/b-catenin signalling pathway in A375 + WLS Sh lung metastasis.

Answer 16: Given that there are 19 different WNTs, more than a dozen different WNT receptors, and numerous kinases , ubiquitin ligases, transcription factors and scaffolding proteins known to be involved in WNT signalling (See " The WNT homepage" http://www.stanford.edu/group/nusselab/cgi-bin/wnt/), we believe that analysing the components of WNT signalling in spontaneous lung metastases is better suited for a follow-up study.

Referee #3 Comment 17: Is WLS normally produced in the lung?

Answer 17: Yes, WLS is expressed in the human lung tissue according to the Gene Card database (www.genecards.org; GeneNotes, Shmueli et al, Comptes Rendus Biologies, 2003; Yanai et al, Bioinformatics, 2005).

Referee #3 Comment 18: What consequence does WLS Sh have in a melanoma cell line with a β-catenin mutation that does not normally form lung metastases? The reverse experiment could also be performed.

Answer 18: We attempted to co-express an activated form of β-catenin along with either *WLS* or control shRNAs in A375 cells, but were surprised to find that β-catenin results in poor viability in these cells . This result is consistent with our previously published studies showing that activating WNT/β-catenin decreases melanoma cell viability (Biechele et al, Science Signaling, 2012; Chien et al, PNAS, 2009). We also transduced Mel 501 human melanoma cells, which harbour an activating mutation in BRAF V600E and an activating mutation in β-catenin (S37F) (Rubinfeld et al, Science, 1997) with WLS and control shRNAs. Unexpectedly, WLS shRNA reduced Mel 501 cell proliferation in two-dimensional culture, which is in contrast to our observations of WLS function in A375, A2058 and MEL624 cells. Further investigation of blocking WNT secretion in melanoma cells expressing activated forms of β-catenin are perhaps better suited for a separate study considering these complications and the scope of this paper.

We did not perform "the reverse experiment," which we interpret to mean knocking down β-catenin in the context of WLS overexpression. β-catenin acts as both a transcription co-factor and as an important mediator of cell adhesions and adherent junctions (Ghislin et al, Pigment cell & Melanoma Research, 2011), therefore, the results of such experiments would be difficult to interpret.

Referee #3 Comment 19: The authors should pay attention to basic errors, such as the misspelling of "crystal" as "chrystal", for example. They should also try to express themselves more concisely in the text. For instance, the following is from the legend of figure 3A. "A375 cells were embedded at single cell density in 0.4% soft agar. Colonies were stained with crystal violet solution. Pictures were taken by 10X object (left) and colony numbers were quantified (right). Scale bars are 1 mm. Crystal violet staining of colonies from cells stably expressing GFP or WLS-GFP after 3 weeks of growth in soft agar". This, and similarly complex phrases elsewhere in the text, should be simplified.

Answer 19: We have corrected this spelling error. We have also simplified complex sentences as much as possible.

Referee #3 Comment 20: In Figure 3D, the authors do not comment on the effects of GAPDH shRNA on the growth or production/localisation of WNT5A/B proteins in A375 melanoma cell lines. They should comment on these aspects.

Answer 20: *GAPDH* shRNA does not regulate melanoma cell proliferation (Figure 3A), but does occasionally increase the amount of WNT5A/B in the media relative to cell lysates (Figure S2A). We have added the following statements to the results section to address this issue:

"Occasionally, we observed elevated WLS and increased levels of secreted WNT5A/B in cells transduced with *GAPDH* shRNA, however these effects were not robust (Fig S2A of Supporting Information). "

2nd Editorial Decision 17 September 2012

Thank you for the submission of your revised manuscript "WLS inhibits melanoma cell proliferation through the -catenin signaling pathway and induces spontaneous metastasis" to EMBO Molecular Medicine. We have now received the reports from the reviewers who were asked to re-review your manuscript.

As you will see, the Reviewers acknowledge that the manuscript was significantly improved during revision. However, both still raise some concerns that should be convincingly addressed.

On a more editorial note, please address the points raised below:

- For experiments involving human subjects the submission must include a statement that informed consent was obtained from all subjects and that the experiments conformed to the principles set out in the WMA Declaration of Helsinki [http://www.wma.net/en/30publications/10policies/b3/] and the NIH Belmont Report [http://ohsr.od.nih.gov/guidelines/belmont.html]. Please see our Guide to Authors for further information and provide the necessary information in the respective Material and Methods part.

- For Research Articles and Reports submitted to EMBO Molecular Medicine reporting experiments on live vertebrates and/or higher invertebrates, the corresponding author must confirm that all experiments were performed in accordance with relevant guidelines and regulations. The manuscript must include a statement in the Materials and Methods identifying the institutional and/or licensing committee approving the experiments, including any relevant details.

- The description of all reported data that includes statistical testing must state the name of the statistical test used to generate error bars and P values, the number (n) of independent experiments underlying each data point (not replicate measures of one sample), and the actual P value for each test (not merely 'significant' or 'P < 0.05').

- We noted that you included unpublished data in your point-by-point response. These would be published in a Peer Review Process File. Please let us know whether you agree with their publication, if you would like to delete the data from the file or if you would like to opt-out of having the PRPF published.

- In addition, we noted that the resolution of panels in Figure 4E and 5B is rather low. Please include higher resolution pictures.

I look forward to reading a new revised version of your manuscript as soon as possible.

Yours sincerely,

Editor
EMBO Molecular Medicine

***** Reviewer's comments *****

Referee #1:

Overall, the authors have satisfactorily the reviewer's questions. The lack of strong statistical evidence (Reviewer Table 1) should be acknowledged in the manuscript.

Referee #3:

The authors improved their manuscript and answered convincingly my major issues.

Comment #5
The information given by the authors should be added in the manuscript (in figure legends for instance).

Comment #10
Obviously double immunostaining on the same sections would have been more convincing. Evaluating, the technical difficulties to perform such experiments, the authors bring an appropriate argument for the time being.

Comment #17
This information could be included in the main text or in the supplemental information.

Comment #18
I fully disagree with this argument for not performing the experiments. However, at this point it is not fully required for this article.

2nd Revision - Authors' Response 20 September 2012

Response to editor's comments:

Editor's comment 1:

- For experiments involving human subjects the submission must include a statement that informed consent was obtained from all subjects and that the experiments conformed to the principles set out in the WMA Declaration of Helsinki [http://www.wma.net/en/30publications/10policies/b3/] and the NIH Belmont Report [http://ohsr.od.nih.gov/guidelines/belmont.html]. Please see our Guide to Authors for further information and provide the necessary information in the respective Material and Methods part.

Answer 1: We included this information in the Materials and Methods as follows:

"All studies involving human subjects were approved by an institutional review board under the supervision of the University of Washington Human Subjects Division (protocol #26161). The experiments performed conform to the principles set out in the WMA Declaration of Helsinki and the NIH Belmont Report. "

Editor's comment 2:

- For Research Articles and Reports submitted to EMBO Molecular Medicine reporting experiments on live vertebrates and/or higher invertebrates, the corresponding author must confirm that all experiments were performed in accordance with relevant guidelines and regulations. The manuscript must include a statement in the Materials and Methods identifying the institutional and/or licensing committee approving the experiments, including any relevant details.

Answer 2: We included this information in the Materials and Methods as follows:

"All animal studies were performed using Institutional Animal Care and Use Committee protocols as approved by a review board at the University of Washington."

Editor's comment 3:

- The description of all reported data that includes statistical testing must state the name of the statistical test used to generate error bars and P values, the number (n) of independent experiments underlying each data point (not replicate measures of one sample), and the actual P value for each test (not merely 'significant' or 'P < 0.05').

Answer 3: We changed the manuscript accordingly. However, exact P values were only available in the parametric tests (t-tests) but not in ANOVA in the GraphPad Prism program, which we used to do statistical analysis.

As noted in the support documentation for the GraphPad Prism 5.0 suite (full text available at http://www.graphpad.com/support/faqid/189/), the value of precise P-values with multiple comparisons such as with ANOVA is questionable:

"When you make several comparisons at once, there is a huge distinction between P values and statistical hypothesis testing. The methods of statistical hypothesis testing can be adjusted to account for the fact you are making multiple comparisons at one time. When you make one comparison, you usually set alpha to 5% which means: If the null hypothesis is true, there is a 5% chance of ending up with a 'statistically significant' result just by chance. When you are making multiple comparisons, you can set things up so that 5% value applies to the entire family of

comparisons. The 5% is said to be the family-wise error rate. This means that if all the null hypotheses were true, there is a 5% chance that one or more of the differences will be 'statistically significant' simply due to random variation, leaving a 95% chance that all the comparisons will be 'not significant'.

Reporting an individual P value, by its very nature, is a way to express the strength-of-evidence for that one comparison. It doesn't really make a lot of sense to compute individual P values.

Prism 5 let you choose the significance level (0.05, 0.01, or 0.001). Whatever significance level you choose, it also displays asterisks to denote the smallest of those significance levels at which the comparison is statistically significant."

Note that our analysis can still distinguish between "significant" ($p<0.05$), "very significant" ($p<0.01$) and "extremely significant" ($p<0.001$). This analysis appears to be consistent with recently published manuscripts in EMBO Molecular Medicine using the GraphPad Prism suite:

example 1: http://www.ncbi.nlm.nih.gov/pmc/articles/PMC3306554/

example 2: http://www.ncbi.nlm.nih.gov/pmc/articles/PMC3306555/

example 3: http://onlinelibrary.wiley.com/doi/10.1002/emmm.201100159/full .

Therefore, we believe that our statistical tests meet the requirements in EMBO Molecular Medicine.

Editor's comment 4:

- We noted that you included unpublished data in your point-by-point response. These would be published in a Peer Review Process File. Please let us know whether you agree with their publication, if you would like to delete the data from the file or if you would like to opt-out of having the PRPF published.

Answer 4: We would prefer to delete the data from the PRPF.

Editor's comment 5:

- In addition, we noted that the resolution of panels in Figure 4E and 5B is rather low. Please include higher resolution pictures.

Answer 5: We replaced the panels accordingly.

Response to reviewer's comments.

Referee #1 (Remarks): Overall, the authors have satisfactorily the reviewer's questions. The lack of strong statistical evidence (Reviewer Table 1) should be acknowledged in the manuscript.

Answer: Thank you very much. We included a sentence in the result section as follows:

"Although the difference in the proportions of nevi and melanomas expressing WLS do not reach statistical significance (Fisher's Exact, P=0.1255) due to the limited sample size, these results are consistent with our finding that expression of WLS transcripts is reduced in melanoma samples in comparison to benign skin tissues (Fig 1A and 1B)."

Referee #3 (Remarks): The authors improved their manuscript and answered convincingly my major issues.

Answer: Thank you very much.

Comment #5

The information given by the authors should be added in the manuscript (in figure legends for instance).

Answer: We added this information in the figure legend of F1A and B as follows:

"Tumour samples were comprised of more than 95% tumour cells."

Comment #10

Obviously double immunostaining on the same sections would have been more convincing. Evaluating, the technical difficulties to perform such experiments, the authors bring an appropriate argument for the time being.

Answer: Thank you.

Comment #17

This information could be included in the main text or in the supplemental information.

Answer: We included this information in the main test of result section as follows:

"We also examined the lungs of the animals used in this study for the presence of metastatic lesions, which express endogenous Wls according to the Gene Card database (www.genecards.org)."

Comment #18

I fully disagree with this argument for not performing the experiments. However, at this point it is not fully required for this article.

Answer: Thank you.

© EMBO
