# Supplementary material for: WLS inhibits melanoma cell proliferation through the β-catenin signalling pathway and induces spontaneous metastasis
Source: EMBO Mol Med. 2012 Nov 6;4(12):1294–307. doi: 10.1002/emmm.201201486 (PMC3531604; doi:10.1002/emmm.201201486)
Supplement: Supplementary file 2 [file emmm0004-1294-SD2.pdf]

WLS inhibits melanoma cell proliferation through the β-catenin signaling pathway and induces spontaneous metastasis

Authors: Pei-Tzu Yang, Jamie N. Anastas, Rachel A. Toroni, Michi M. Shinohara, Jamie M. Goodson, Anja K. Bosserhoff, Andy J. Chien, and Randall T. Moon.

Supporting Information content Page 1

Supporting Information materials and methods Page 2
- siRNA sequences
- qPCR primer sequences

Supporting Figures
- Supporting Figure S1. Validation of WLS antibody specificity in IHC staining. Page 3
- Supporting Figure S2. Increasing or decreasing WLS expression disrupts endogenous WNT secretion. Page 4
- Supporting Figure S3. Reduced WLS does not affect primary tumor growth in xenograft studies nor cell growth in vitro at high density. Page 5
- Supporting Figure S4. Low levels of WLS are associated with distant metastases in melanoma patients. Page 6
- Supporting Figure S5. Reduced WLS does minimally affect cell invasion and migration in vitro. Page 7
- Supporting Figure S6. Validation of WNT siRNAs in A375 cells. Page 8
- Supporting Figure S7. WLS is a WNT/β-catenin target. Page 9
- Supporting Figure S8. Validation of cell counting by high content imaging using the In Cell Analyzer 2000. Page 10
- Supporting Figure S9. Un-cropped immunoblot images. Page 11

Supporting Tables
- Supporting Table S1. Summary of WLS staining in benign nevi and cutaneous melanomas. Page 12
- Supporting Table S2. Summary of WLS expression in the microarray studies comparing paired primary and metastatic melanoma samples. Page 13

Supporting Materials and Methods

siRNA sequences (sense)
β-catenin siRNA: CUAUCUGUCUGCUCUAGUA[dT][dT]
WLS siRNA: AACUCACGAAUCCCUUCUACAGUAU[dT][dT]
VPS35 siRNA: CUGUAGGGAUGCUUUGGCUTT[dT][dT]
WNT2B siRNA: CCCUCAUGAACUUACAUAA[dT][dT]
WNT5A siRNA 1: GGUUGUAAUUGAAGCCAAU[dT][dT]
WNT5A siRNA 2: GGACUUUCUCAAGGACAGA[dT][dT]
WNT9A siRNA: GCACAAGUAUGAGACGGCA[dT][dT]
WNT10B siRNA: GCACGAAUGCGAAUCCACA[dT][dT]
WNT11 siRNA: CUCUGGAAAUGAAGUGUAA[dT][dT]
WNT16 siRNA: GGCAGAGAAUGCAACCGUA[dT][dT]

Quantitative PCR primer sequences
AXIN2: F: CTCCCCACCTTGAATGAAGA, R: TGGCTGGTGCAAAGACATAG
GAPDH: F: TGAAGGTCGGAGTCAACGGA, R: CCATTGATGACAAGCTTCCCG
RN18S1: F: GGTGAAATTCTTGGACCGGC, R: GACTTTGGTTTCCCGGAAGC
WLS: F: TATCTGGACTACAGACATTGG, R: CTTCCGTTACCTGACTAACG
WNT1: F: ATGAACCTTCACAACAACGA, R: TTGGGCGATTTCTCGAAGTA
WNT2: F: CAGGGTGATGTGCGATAATG, R: CAAAGGCAGATTCCCGACTA
WNT2B/13: F: ATGGATCCGAGAGTGTCAGC, R: GCGACCACAGCGGTTATTAT
WNT3: F: TGGAACTGCACCACCATAGA, R: GGACACTAACACGCCGAAGT
WNT3A: F: GTTTGGTGGGATGGTGTCTC, R: GCGCTGTCGTACTTGTCCTT
WNT4: F: GCATCTCAGAGGAGGAGACG, R: AGAGATGGCGTACACGAAGG
WNT5A: F: TAAGCCCAGGAGTTGCTTTG, R: TGACATCTGAACAGGGTTATTCAT
WNT5B: F: TCTGACAGACGCCAACTCCT, R: GCATTCCTTGATGCCAGTCT
WNT6: F: GGACGAGAAGTCGAGGCTCT, R: CGAAATGGAGGCAGCTTCT
WNT7A: F: GCCTGGACGAGTGTCAGTTT, R: CTGGCCTTGCTTCTCTTTGT
WNT7B: F: GTTACGGCATCGACTTCTCC, R: GTTGGGCGACTTCTCAATGT
WNT8A: F: GCCTATCTGACCTACACGAC, R: TCTCTGGTAGCACTTCTCAG
WNT8B: F: GGGCTTTGAGAATTCCATCC, R: GTAAATCAGGTAAGCCTTTGGAC
WNT9A/14: F: TGCCTTCCTCTATGCCATCT, R: CTTGTGCTTCAGATGCTTGC
WNT9B/15: F: GCTGGAACTGTAGCCTGGAG, R: ACCCCAGGAAGTTGCTCAG
WNT10A: F: CGAATGCCAACACCAATTCA, R: GCAGTGCATCCAGTTGTAAG
WNT10B: F: ATGACATGGACTTTGGAGAG, R: CGGTTGTGGGTATCAATGAA
WNT11: F: CACTGAACCAGACGCAACAC, R: ATACACGAAGGCCGACTCC
WNT16: F: CGAGAGATGGAACTGCATGA, R: TCTGTCATGTTGCCTGCACT
hsa-miR-200a-5p: ATCTTACCGGACAGTGCTGGA

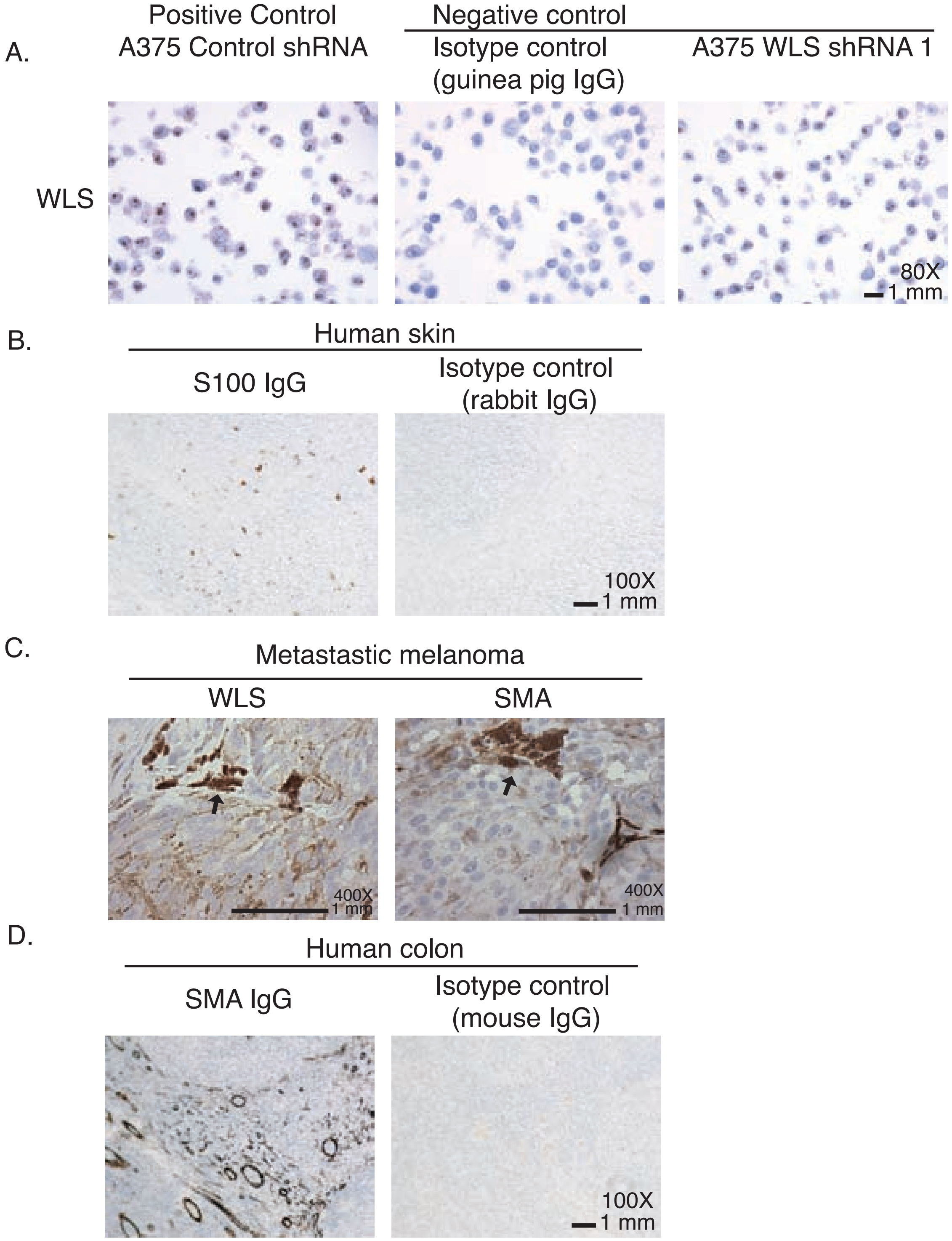

Supporting Figure S1. Validation of WLS antibody specificity in IHC staining. (A) A375 cells were fixed in formaldehyde, embedded in paraffin, and sectioned prior to immunohistochemistry using the WLS antibody. Positive staining (brown color) is observed in samples that were stained with our WLS antibody, but not with a control IgG antibody (compare left and center panels). WLS shRNA reduced the signal observed with WLS antibody (compare left and right panels), indicating that the signal is specific. (B) Normal human skin stained using either antibodies to detect the melanocyte marker, S100 (left panel, brown color), or an isotype control (right panel). (C) Higher magnification of Fig 1I. Strong WLS staining in the smooth muscle cells, indicated by an arrow in the left panel. Smooth muscle cells were marked by smooth muscle actin (SMA) in the adjacent serial section, indicated by an arrow in the right panel. (D) Human colon stained with a SMA antibody (left panel, brown color) or an isotype control (right panel).

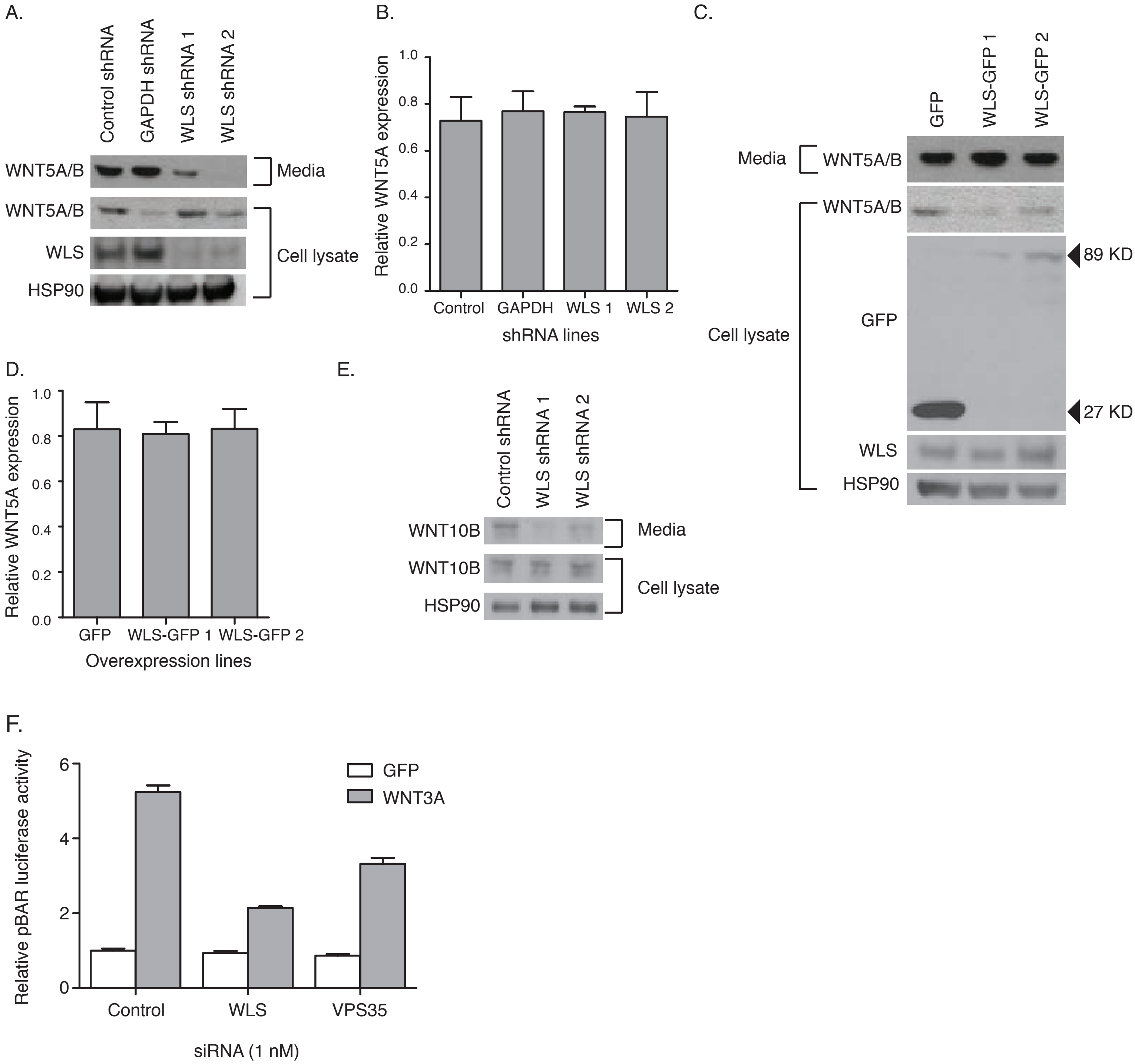

Supporting Figure S2. Increasing or decreasing WLS expression disrupts endogenous WNT secretion. (A) Immunoblot analysis of endogenous WNT5A, WLS and HSP90 in A375 cells transduced with WLS, GAPDH and control shRNAs. Protein samples were collected from media and cell lysates. (B) Quantification of WNT5A mRNA in A375 cells transduced with shRNA targeting WLS and GAPDH or a non-silencing control. (C) Immunoblot analysis of WNT5A, GFP, WLS and HSP90 in A375 cells stably expressing WLS-GFP or GFP. Protein samples were collected from media and cell lysates. Arrows indicate predicted molecular weight of WLS-GFP (89KD) and GFP (27KD). (D) Quantification of WNT5A mRNA in A375 cells transduced with WLS-GFP or GFP. (E) Immunoblot analysis of endogenous WNT10B and HSP90 in A375 cells transduced with WLS and control shRNAs. (F) Normalized WNT/β-catenin transcriptional reporter (BAR-luciferase) activity in A2058 cells transfected with WNT3A or GFP constructs, as well as siRNA targeting WLS, VPS35 or control.

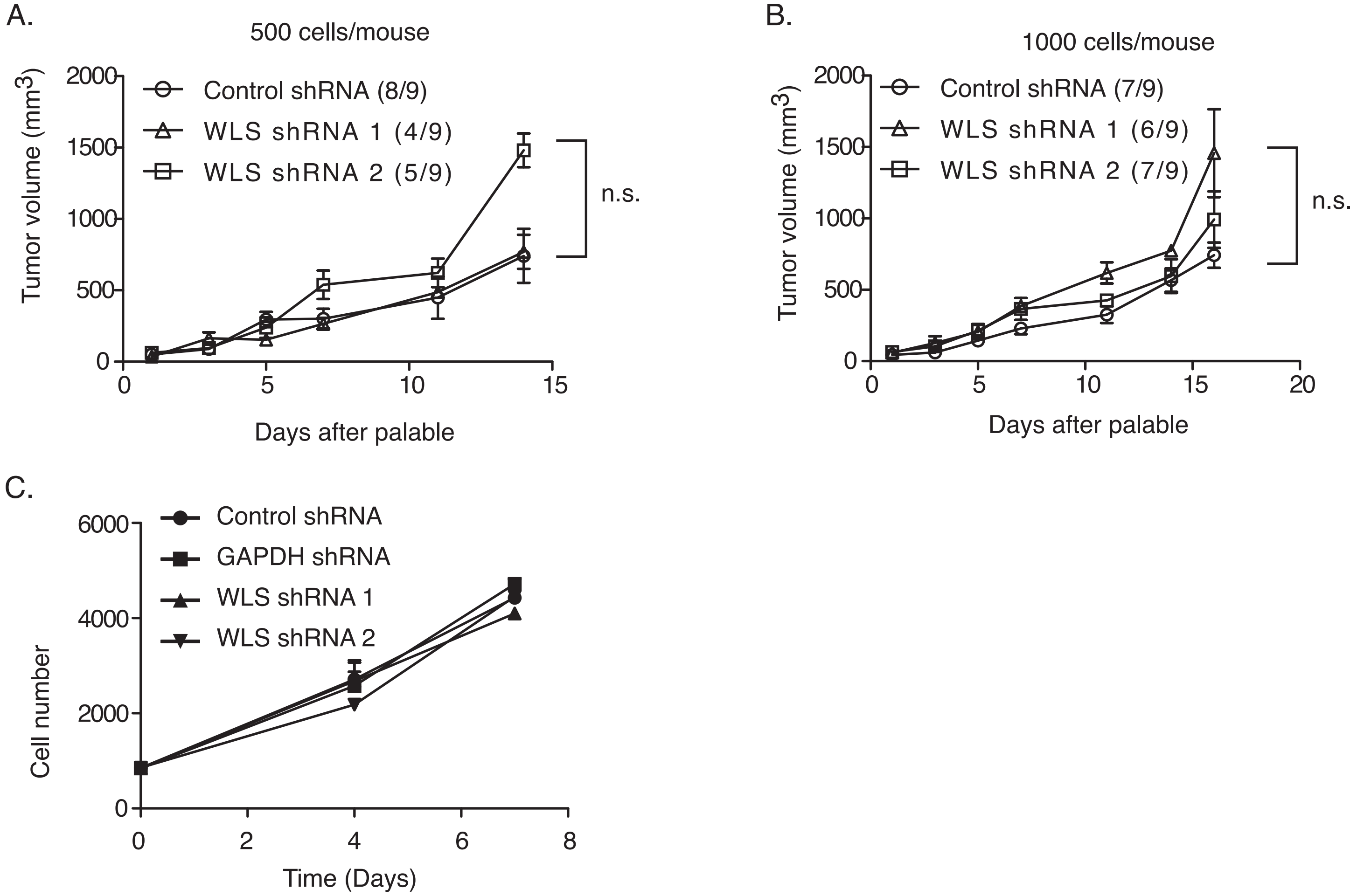

Supporting Figure S3. Reduced WLS does not affect primary tumor growth in xenograft studies nor cell growth in vitro at high density. (A and B) Growth curves of tumors derived from A375 stable lines transduced with control or WLS shRNAs. Tumor growth curves were started (tine 0) when tumors became palpable. The fractions of animals that developed primary tumors at 40 days following initial cell injections are indicated in parentheses. (A) Xenograft tumor growth curves generated from mice that were injected with 500 cells, P=0.0924. (B) Xenograft tumor growth generagted curves from mice that were injected with 1000 cells, P=0.1364. (C) Growth curves of A375 cells transduced with shRNA against WLS, GAPDH or control. Cells were seeded at high density (2500 cells/cm^2).

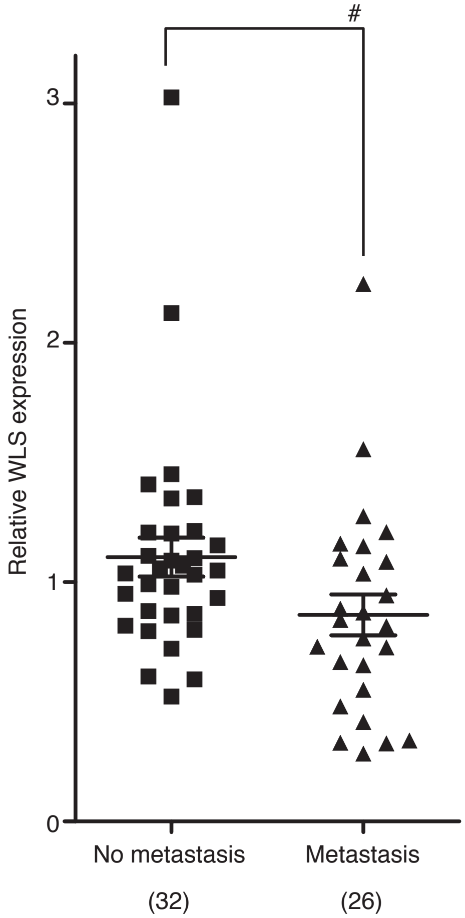

Supporting Figure S4. Low levels of WLS are associated with distant metastases in melanoma patients. Summary of WLS expression levels (probe# A24_256380) in a microarray dataset published by Winnepenninckx et al. (2006). This dataset compares gene expression in primary cutaneous melanoma tumors that were categorized according to the presence of distant metastases. In this study, a subset of patients developed metastatic tumors within four years after diagnosis (n=32) and a subset of patients did not (n=26), #P=0.0471.

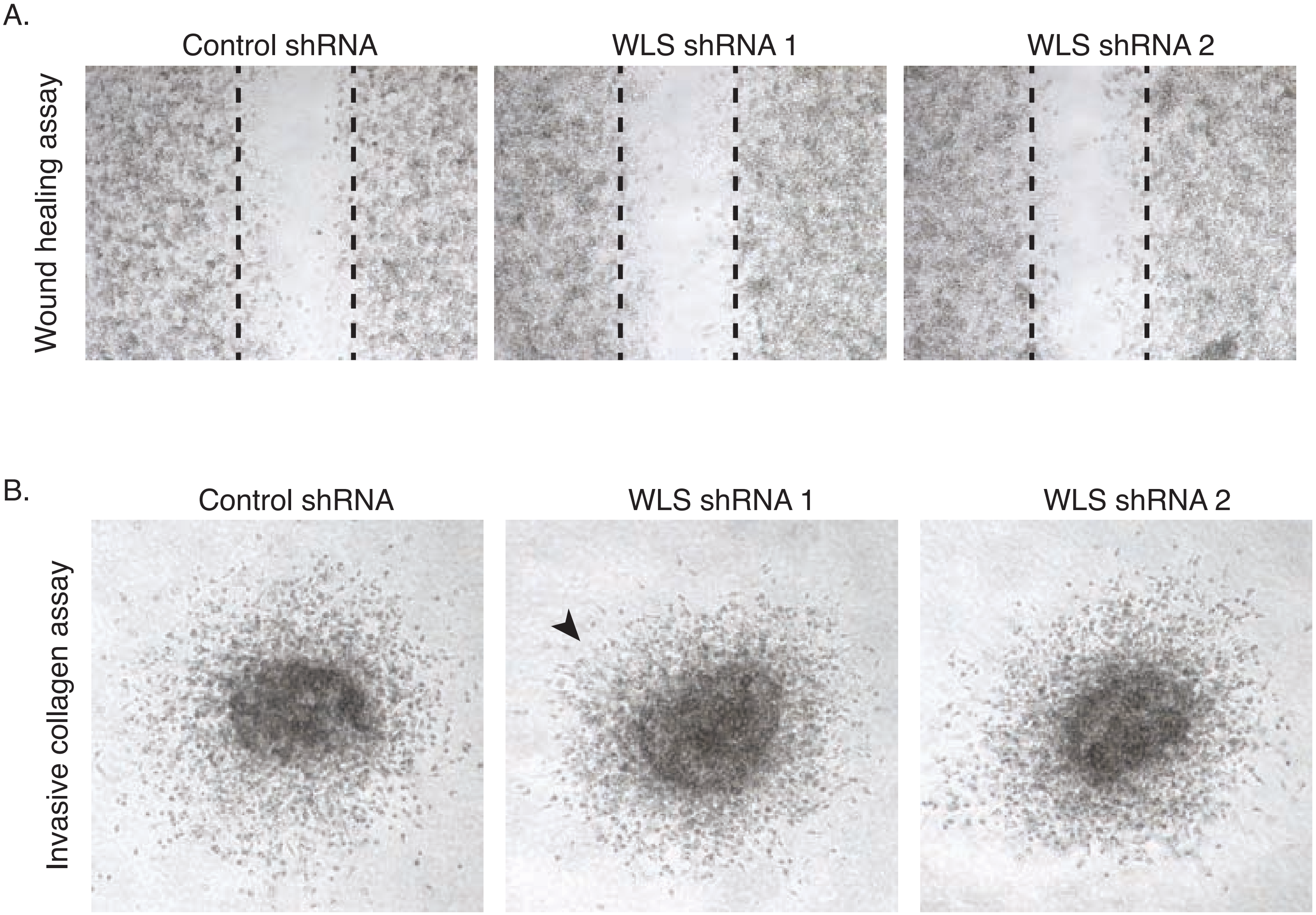

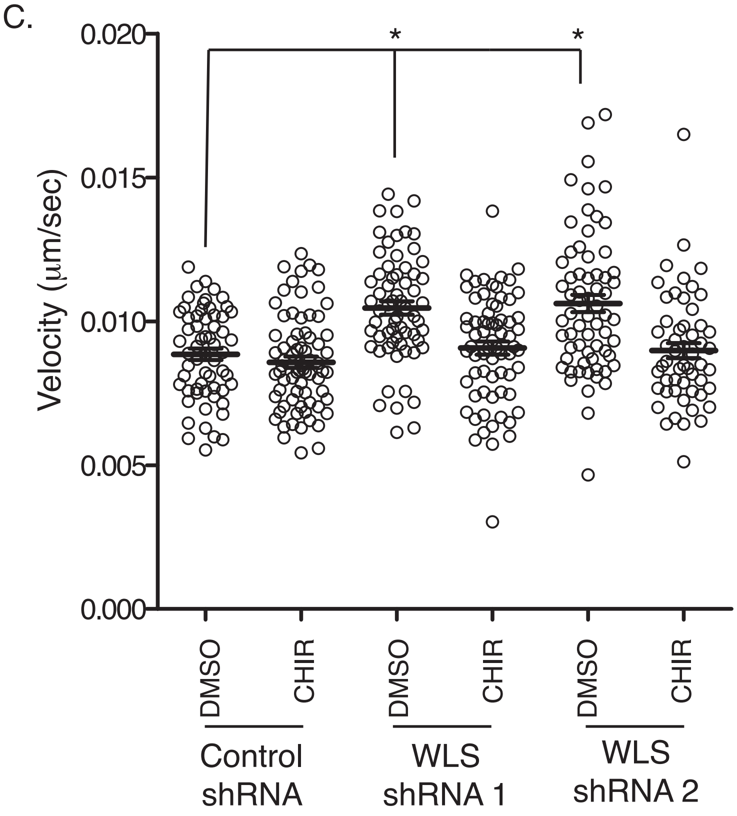

Supporting Figure S5. Reduced WLS does minimally affect cell invasion and migration in vitro. (A) Wound healing assays were performed with A375 cells transduced with WLS or control shRNAs. Pictures were taken immediately and 24 hours after wounding confluent cell monolayers. Dotted lines indicate the scratch at time point 0. (B) The invasiveness of A375 cells expressing control or WLS sRNA was determined by conducting spheroid assays. First, 5000 A375 cells were cultured as spheroids in low attachment plates for 72 hours. Spheroids were then embedded in type I collagen. Pictures were taken 24 hours after embedding. (C) A375 cells were plated on type I collagen and the migration of individual cells was determined by analysis of video microscopy data. More than 75 cells per condition were tracked in three individual experiments. In some cases, cells were pre-treated with either DMSO or 0.5 μM CHIR99021 (CHIR) for 24 hours before conducting the assays. Cells were similarly treated with these compounds during the migration experiments. Migration velocity was calculated by dividing total path length by time (μm/hour), $P<0.01$.

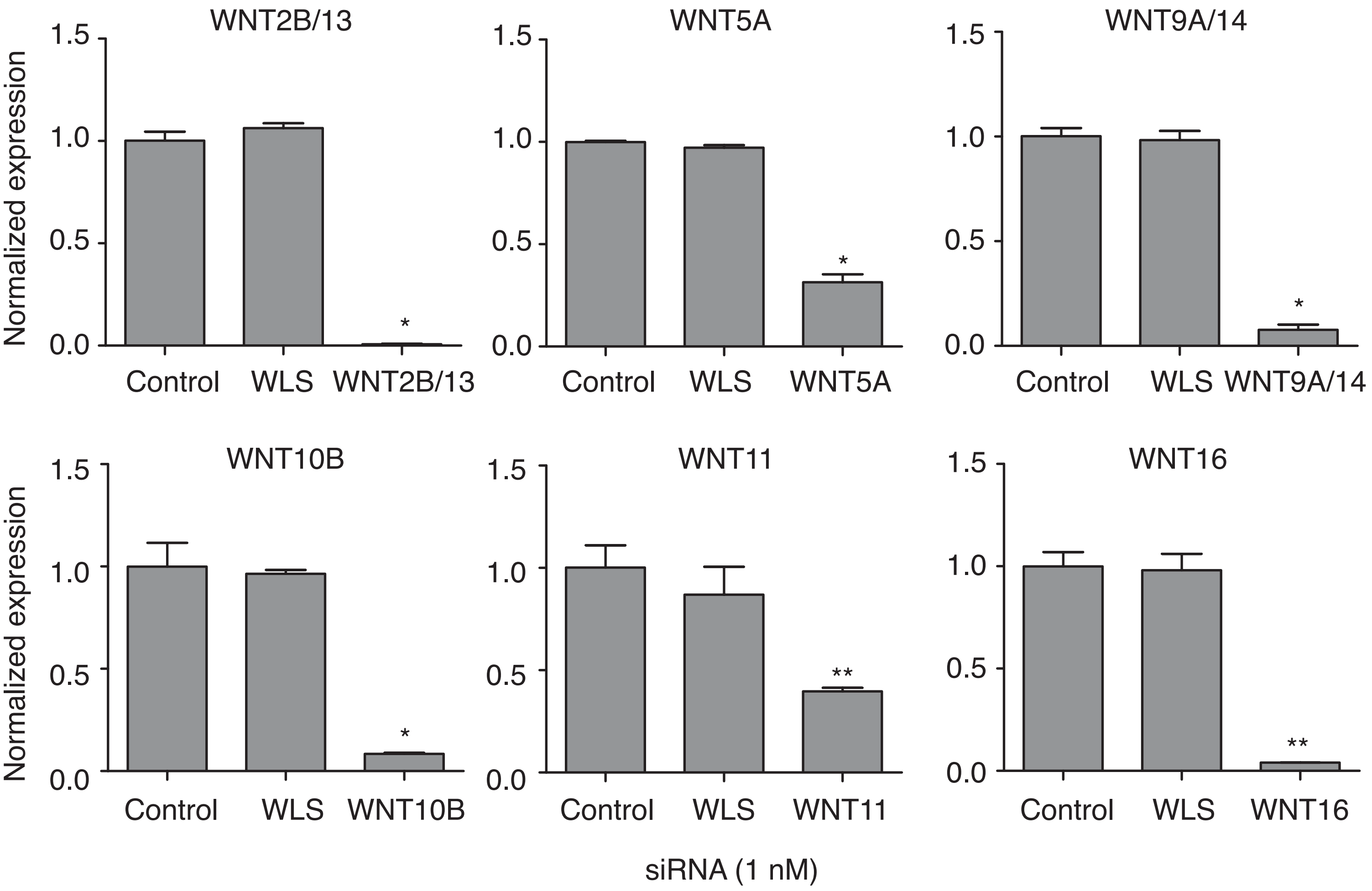

Supporting Figure S6. Validation of WNT siRNAs in A375 cells. Expression of WNT ligands following siRNA transfection quantified by qPCR. WNT expression values were normalized to control siRNA set at 1.0. RNA samples were collected 72 hours post transfection with WLS or WNT siRNAs, *P<0.0001, **P<0.005.

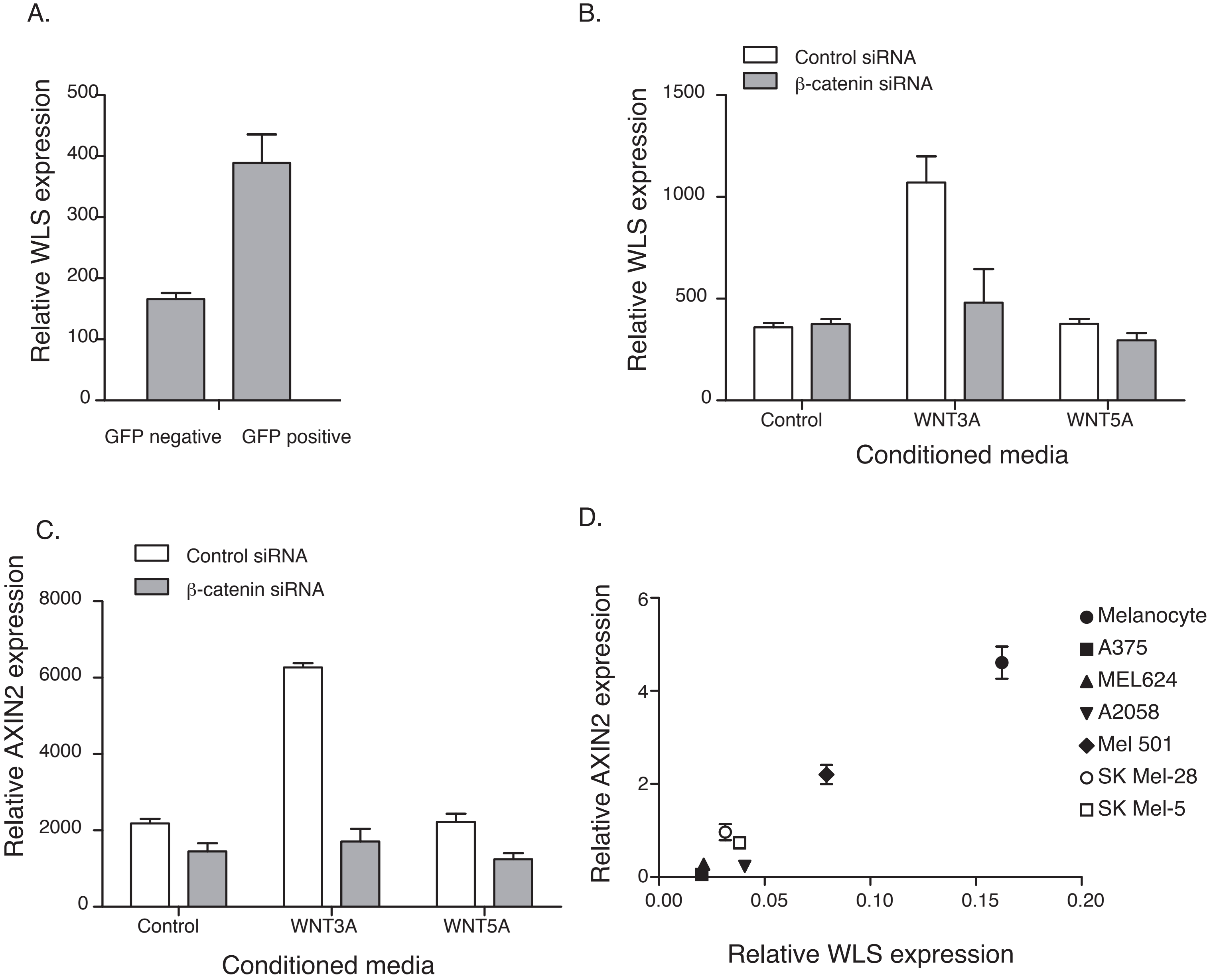

Supporting Figure S7. WLS is a WNT/β-catenin target. (A) Relative WLS expression normalized to GAPDH in A375 cells transduced with a β-catenin activated reporter (BAR) driving Venus GFP. GFP positive cells with active β-catenin-dependent transcription and GFP negative cells were obtained by fluorescence activated cell sorting. (B) Relative WLS expression in A375 cells transfected with siRNA against β-catenin/CTNNB1 or a control siRNA and treated with 10% WNT3A, WNT5A or control conditioned-media. RNA was collect 72 hours after transfection and WLS expression was quantified by qPCR. (C) Relative expression of the WNT/β-catenin target, AXIN2 quantified in the same samples described in (B). (D) Positive correlation between the expression of the WNT/β-catenin target gene, AXIN2 and the expression of WLS in human melanocytes and melanoma cells, Spearman R=0.8035, P=0.0366.

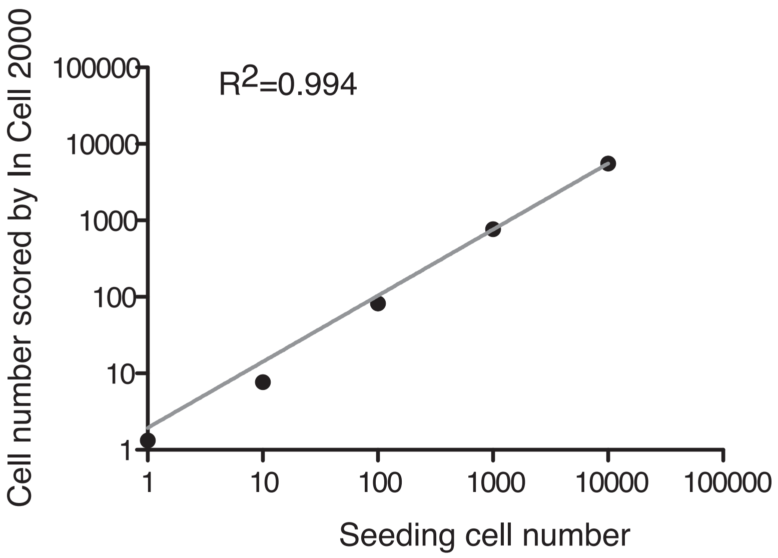

Supporting Figure S8. Validation of cell counting by high content imaging using the In Cell Analyzer 2000. The number of cells seeded (x-axis) are compared to the number of cells counted (y-axis), R2=0.994.

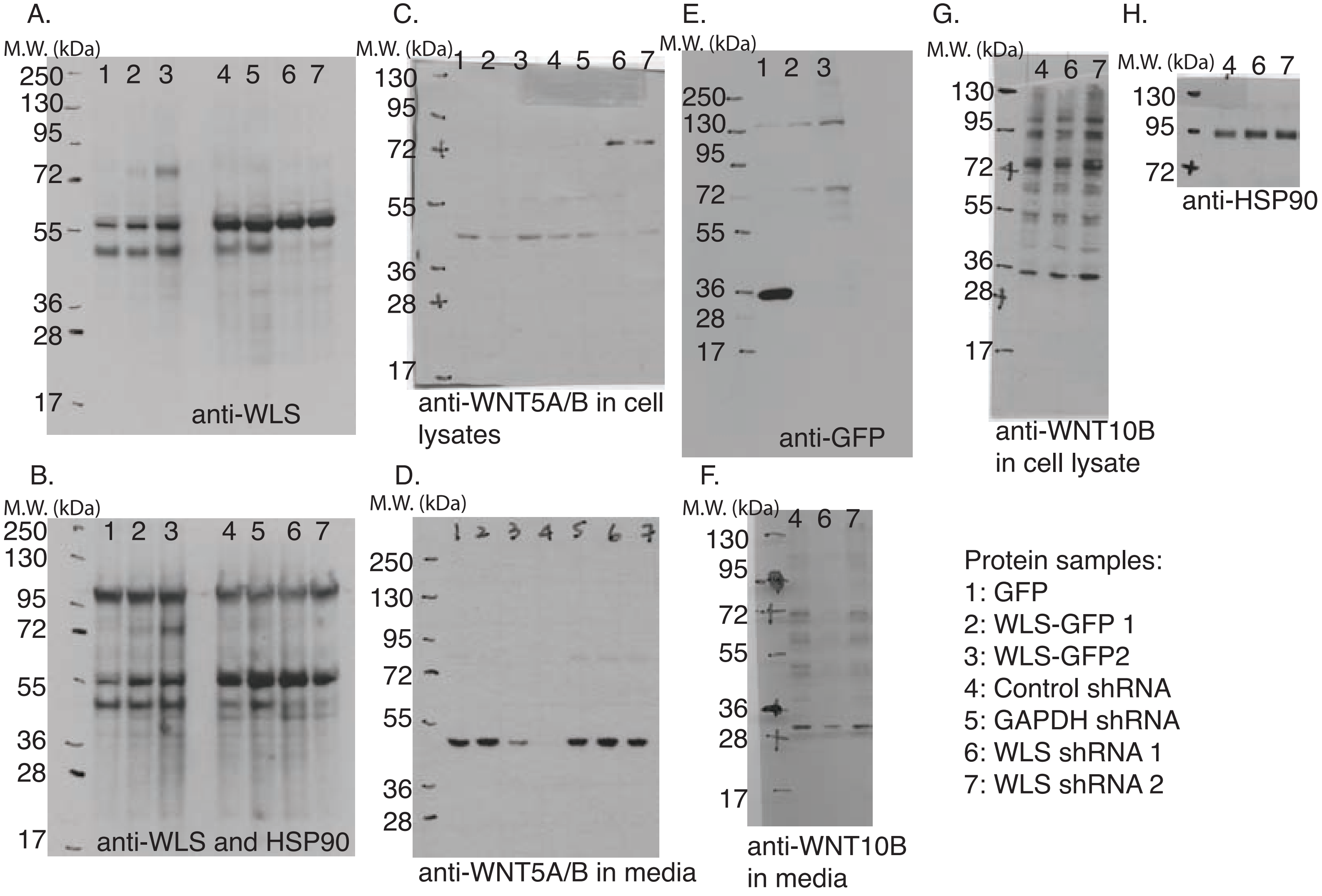

Supporting Figure S9. Un-cropped immunoblot images. Original immunoblot images used to generate panels in Supporting Figure S2.

Supporting Table S1. Summary of WLS staining in benign nevi and cutaneous melanomas.

Figure panel	Tissue	Specimen	% staining*	Intensity**	Notes
	nevi	03-15650	1	2	staining of scattered dermal cells
	nevi	03-1724A	1	2	staining of intraepidermal melanocytes
	nevi	03-1885B	1	2	staining of superficial dermal componenet and scattered dermal cells
	nevi	03-15778	1	1	staining of superficial dermal cells
	nevi	03-6808	3	2	staining of superficial dermal componenet and scattered dermal cells
	nevi	03-1032A	1	2	stianing of junctional and superficial dermal nests
	nevi	03-1032B	1	2	stianing of junctional and superficial dermal nests
	nevi	03-787	1	1	staining of superficial dermal cells
Figure 1D&E	nevi	03-1032	1	2	staining of intraepidermal nests
	nevi	03-916	0	0	
	primary melanoma	03-19604	1	2	strong staining globules in large, atypical cells
	primary melanoma	03-20813	1	1	strong staining dendritic interstitial cells, eccrine coils and smooth muscle cells
	primary melanoma	03-24545	2	2	staining of intraepidermal componenet and dermal components
	primary melanoma	03-26900	1	2	
	primary melanoma	04-2669	0	0	
	primary melanoma	04-5	0	0	
Figure 1F&F'	primary melanoma	01-25871	1	2	staining of intraepidermal componenet
	primary melanoma	01-25957	1	1	granular staining
	primary melanoma	03-16065	0	0	
	primary melanoma	03-20667	1	1	
	primary melanoma	03-16376A	0	0	
	primary melanoma	03-16376B	0	0	
	primary melanoma	03-16376C	0	0	
	primary melanoma	03-16376D	0	0	
	primary melanoma	01-15305	0	0	
	primary melanoma	03-17503	1	2	
	primary melanoma	03-27186	0	0	
	primary melanoma	01-17897	1	2	
Figure 1G&G'	metastastic melanoma	03-16262	3	2	strong staining dendritic interstitial cells, eccrine coils and smooth muscle cells
	metastastic melanoma	02-22435	1	2	

* 0=negative 1=0-25% 2=26-50% 3=51-75% 4=76-100%

** 0=negative 1=weak 2=strong

Publication	Metastatic melanoma sample size	Primary melanoma sample size	Fold change§	P value
Winnepennickx et al., 2007	9	9	1.07	N/A¶
Valsesia et al., 2011	11	11	0.10	N/A¶
Pasmanik-Chor et a., 2011	4	4	0.99	P=0.7021

Supporting Table S2. Summary of *WLS* expression in the microarray studies comparing paired primary and metastatic melanoma samples. Fold change is calculated by dividing the value of *WLS* expression in metastatic tumors by the value of *WLS* expression in paired primary tumors.
